# Noncanonical mitochondrial unfolded protein response impairs placental oxidative phosphorylation in early-onset preeclampsia

**DOI:** 10.1073/pnas.1907548116

**Published:** 2019-08-22

**Authors:** Hong Wa Yung, Francesca Colleoni, Emilie Dommett, Tereza Cindrova-Davies, John Kingdom, Andrew J. Murray, Graham J. Burton

**Affiliations:** ^a^Centre for Trophoblast Research, University of Cambridge, Cambridge CB2 3EG, United Kingdom;; ^b^Department of Physiology, Development and Neuroscience, University of Cambridge, Cambridge CB2 3EG, United Kingdom;; ^c^Department of Obstetrics and Gynaecology, Mount Sinai Hospital, University of Toronto, Toronto, ON, Canada M5G 1X5

**Keywords:** preeclampsia, unfolded protein response, mitochondria

## Abstract

Preeclampsia (PE) is a dangerous complication of pregnancy, especially when it presents at <34 wk of gestation (PE < 34 wk). It is a major cause of maternal and fetal morbidity and mortality and also increases the risk of cardiometabolic diseases in later life for both mother and offspring. Placental oxidative stress induced by defective placentation sits at the epicenter of the pathophysiology. The placenta is susceptible to activation of the unfolded protein response (UPR), and we hypothesized this may affect mitochondrial function. We first examined mitochondrial respiration before investigating evidence of mitochondrial UPR (UPR^mt^) in placentas of PE < 34 wk patients. Reduced placental oxidative phosphorylation (OXPHOS) capacity measured in situ was observed despite no change in protein or mRNA levels of electron transport chain complexes. These results were fully recapitulated by subjecting trophoblast cells to repetitive hypoxia–reoxygenation and were associated with activation of a noncanonical UPR^mt^ pathway; the quality-control protease CLPP, central to UPR^mt^ signal transduction, was reduced, while the cochaperone, TID1, was increased. Transcriptional factor ATF5, which regulates expression of key UPR^mt^ genes including HSP60 and GRP75, showed no nuclear translocation. Induction of the UPR^mt^ with methacycline reduced OXPHOS capacity, while silencing *CLPP* was sufficient to reduce OXPHOS capacity, membrane potential, and promoted mitochondrial fission. CLPP was negatively regulated by the PERK-eIF2α arm of the endoplasmic reticulum UPR pathway, independent of ATF4. Similar changes in the UPR^mt^ pathway were observed in placentas from PE < 34 wk patients. Our results identify UPR^mt^ as a therapeutic target for restoration of placental function in early-onset preeclampsia.

## Significance

Preeclampsia endangers the lives and well-being of mother and baby. The syndrome is associated with placental dysfunction. High demand for energy to support active nutrient transport and hormone production increases placental susceptibility to mitochondrial stress. Here, we investigate mitochondrial activity and explore stress-response pathways in preeclamptic placentas. We demonstrate activation of noncanonical mitochondrial unfolded protein response (UPR^mt^) pathways associated with reduced CLPP, a key protease in UPR^mt^ signalling, that compromises mitochondrial respiration. The changes can be recapitulated in trophoblast cells by hypoxia–reoxygenation. Either activation of UPR^mt^ or knockdown of CLPP can sufficiently reduce mitochondrial respiration. Translation of CLPP is negatively regulated by the endoplasmic reticulum UPR pathway. Understanding mitochondrial stress provides new insights into the pathophysiology of early-onset preeclampsia.

Preeclampsia (PE) is a hypertensive disorder that occurs in 3 to 5% of human pregnancies in developed countries (1) and is a major cause of maternal and neonatal mortality and morbidity. Two subtypes of the disorder are recognized based on the time of clinical onset (2). Early-onset PE (<34-wk gestational age) is typically initiated by defective placentation in otherwise healthy women and is characterized by reduced uteroplacental blood flow that results in an abnormal angiogenic profile in the maternal blood and high systemic vascular resistance. By contrast, in late-onset disease the pathophysiology is thought to center around interactions between normal senescence of the placenta and a maternal genetic predisposition to cardiovascular and metabolic disease (2–4).

In the canonical pathway of placenta-mediated disease, the pathogenesis is triggered by chronic low-grade ischemia–reperfusion injury to the placental villi and is perpetuated by oxidative stress in the trophoblast epithelial compartment in direct contact with the maternal blood (5, 6). This layer shows morphological changes indicative of stress, including distorted microvilli, dilated cisternae of endoplasmic reticulum (ER), and areas of focal necrosis (7). At the molecular level there is accompanying evidence of senescence (8), reduced secretion of the proangiogenic protein placenta growth factor (PlGF), and increased secretion of the sFlt-1 receptor that acts as an antagonist of vascular endothelial growth factor (9). Measurement of these proteins in maternal blood is now central to the clinical diagnosis of the disease (10, 11).

While the molecular basis of these key changes is not fully understood, much is known regarding the effects of oxidative stress on trophoblast cellular functions. Accumulation of oxidatively damaged unfolded/misfolded proteins is potentially toxic to cells and so protective organelle-specific signaling pathways, generically referred to as unfolded protein responses (UPRs), are activated. UPRs are present in all cellular compartments capable of protein synthesis, including the cytoplasm (UPR^cyto^), mitochondria (UPR^mt^), and ER (UPR^ER^). The UPR is a homeostatic mechanism that aims to restore cellular functions or to remove damaged cells (12). Increasing evidence demonstrates cross-talk between the UPR^ER^ and UPR^mt^ (13) acting through mitochondria-associated ER membranes and Ca^2+^ homeostasis. Our group was the first to demonstrate activation of the placental UPR^ER^ in early-onset PE (14). Here, we sought evidence of the UPR^mt^ and its impact on mitochondrial respiration.

In comparison to the UPR^ER^, the signaling pathways involved in the UPR^mt^ are poorly understood (15). The UPR^mt^ is particularly active in cells with high production of reactive oxygen species, high rates of mitochondrial biogenesis, and defective mitochondria (16, 17). The majority of mitochondrial proteins are synthesized in the cytosol, and nascent polypeptides translocate into the matrix (18). They undergo chaperone-assisted folding into their active conformation and assembly into multiprotein units, such as electron transport chain (ETC) complexes (18). An evolutionarily conserved chaperone system that includes HSP60/HSP10 and GRP75/TID1 (also known as mtHSP70/DNAJA3) and proteases is involved in the folding and quality control processes, respectively (19).

HSP60 facilitates folding of nascent polypeptides, while GRP75 binds to misfolded polypeptides, assisting their refolding. During refolding, TID1, a cochaperone, stimulates the ATPase activity of GRP75 (20). The mitochondrial protein degradation machinery is mainly mediated by 2 key quality-control proteases, CLPP and paraplegin, each with different substrate preferences. In *Caenorhabditis elegans*, ClpXP, an AAA+ protease equivalent to mammalian CLPP, is at the core of the UPR^mt^ signaling transduction. This protease degrades misfolded/unfolded proteins into short peptides that are extruded to the cytosol (21), where they activate ATFS-1 (Activating Transcription Factor associated with Stress, also known as ZC376.7). This in turn translocates to the nucleus and facilitates expression of mitochondrial chaperones and proteases (21, 22). While similar transcriptional responses to UPR^mt^ have been described in mammalian cells (23), the pathway sensing unfolded/misfolded proteins is unknown. A recent study identified activating transcription factor 5 (ATF5), a member of the cAMP response-element binding protein (CREB) family, as regulating mitochondrial chaperones HSP60 and GRP75, and proteases CLPP and LONP (24). Furthermore, ATF4, which belongs to the same CREB family and is a downstream effector of the UPR^ER^ PERK/eIF2α pathway, acts as a key regulator of the mitochondrial stress response in mammals (25). These findings illustrate the close interplay between the UPR^ER^ and UPR^mt^. Hence, it is likely they are coactivated under stress conditions. If mitochondrial function is severely impaired, mitophagy is activated to clear damaged organelles (13).

Here, we first characterized the extent of mitochondrial dysfunction in placentas from early-onset PE. Next, we elucidated potential regulatory mechanisms involved in the UPR^mt^ pathway using an in vitro model involving repetitive hypoxia–reoxygenation (rHR) of trophoblast-like BeWo cells (4, 26). We manipulated the UPR^mt^ to investigate its impact on mitochondrial function and relationship with the UPR^ER^ pathway. Finally, we explored activation of the UPR^mt^ pathway in placental samples from preeclamptic patients.

## Results

### Reduction of Oxidative Phosphorylation Capacity in Placentas of Early-Onset Preeclampsia.

**Fig. 1.**
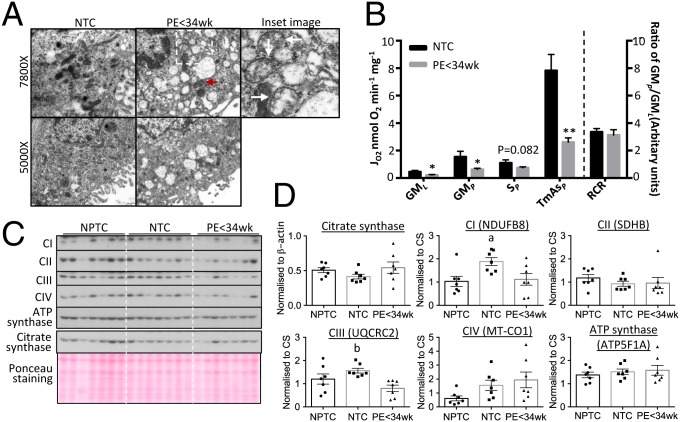
Reduction of OXPHOS capacity in mitochondria with intact ETC complexes subunits in PE < 34 wk placentas. (*A*) Placental mitochondria from PE appear swollen, with distorted cristae and less elongated, more rounded profiles suggestive of a high incidence of fission compared to controls. Red arrowhead indicates normal mitochondrion. (*Inset* ) Illustration of enlarged mitochondria with distorted cristae (arrows). The images were taken at either 5,000× or 7,800×. (*B*) Reduction of mitochondrial OXPHOS capacity in the PE < 34 wk placenta. Respirometry was used to measure activity of ETC complexes after addition of glutamate and malate (GM_*L*_) indicating leak respiration; ADP (GM_*P*_) indicating complex I OXPHOS; rotenone + succinate (S_*P*_) indicating complex II respiration; and TMPD + ascorbate (TmAs_*P*_) corresponding to complex IV respiration. RCR was calculated as the ratio of GM_*P*_:GM_*L*_. Results are presented as mean ± SEM, *n* for NTC = 7 and PE < 34 wk = 12. *P < 0.05; **P < 0.01. (*C* and *D*) No alteration of ETC complex subunit protein levels and constant CS in the PE < 34 wk placenta compared to NPTC. (*C*) Western blots. (*D*) Quantitative data after normalization to CS. Data are presented as mean ± SEM, *n* = 7. a and b indicate significant change (*P* < 0.05) in NPTC vs. NTC and NTC vs. PE < 34 wk, respectively. Two-tailed unpaired Student’s *t* test was used for statistical analysis except in *D* where 1-way ANOVA with Tukey’s multiple comparisons test was employed.

Swelling is a hallmark of mitochondrial dysfunction. Therefore, we first examined placental mitochondrial ultrastructure. Fewer normal elongated, rod-shaped mitochondria and more abnormal swollen profiles with distorted cristae were observed in the syncytiotrophoblast of early-onset preeclamptic (PE < 34 wk) placentas compared with normotensive controls (NTC) (arrows in Fig. 1 *A*, *Inset*). The presence of occasional mitochondria with normal morphology (Fig. 1 *A*, red arrowhead) indicated the latter changes were not fixation artifacts, nor universal. We also observed a larger number of rounded, short mitochondrial profiles in the PE < 34 wk placentas, suggesting possible mitochondrial fragmentation (Fig. 1*A*). These changes were associated with dilation of the ER, indicating loss of homeostasis within the cisternae in the PE < 34 wk placentas.

Next, we evaluated mitochondrial function using respirometry. Previous studies addressing mitochondrial oxidative phosphorylation (OXPHOS) activity in placentas from preeclamptic pregnancies have been limited to either primary trophoblast cells or to isolated mitochondria (27, 28). Instead, we used thawed cryopreserved placental villous samples permeabilized using saponin, in which the mitochondria are retained in their normal cytoplasmic relationships (29). Oxygen consumption was measured using Clark-type oxygen electrodes (29), and 12 PE < 34 wk and 7 NTC placental samples were compared (Table 1). Although normotensive preterm control placentas (NPTC) are, in principle, an ideal control, those available are not stress-free owing to the clinical conditions that triggered spontaneous or iatrogenic preterm delivery. In particular, most preterm placentas have been exposed to ischemia–reperfusion during vaginal delivery, a potent stimulus of oxidative and ER stress (30). Nonlabored caesarean-delivered NPTC placentas from healthy pregnancies are virtually impossible to obtain due to the rarity of indicated nonlabor caesarean delivery at comparable gestation ages to women delivering with PE at <34 wk.

**Table 1.** Clinical characteristics of placentas for respirometry

Characteristics	NTC (*n* = 7)	PE < 34 wk (*n* = 12)	*P* value
Gestational age, wk	39.3 ± 1.2	30.7 ± 1.8	*P* < 0.001
Systolic blood pressure	123 ± 8.9	166.2 ± 11.4	*P* < 0.001
Diastolic blood pressure	79.5 ± 3.3	101.8 ± 7.2	*P* < 0.001
Birth weight, g	3,350 ± 376	1,142 ± 310	*P* < 0.001
Placental weight, g	458 ± 50	185 ± 56	*P* < 0.001

In the presence of malate and glutamate, as substrates for the N-pathway via complex I (GM_*P*_), and with *N*, *N*, *N′*, *N′*-tetramethyl-*p*-phenylenediamine (TMPD) and ascorbate as nonphysiological electron donors for complex IV (TmAs_*P*_), OXPHOS respiration was more than 60% lower (*P* < 0.05 and *P* < 0.01, respectively) in PE < 34 wk placentas compared with NTC (Fig. 1*B*). Additionally, LEAK respiration via the N-pathway (GM_*L*_) was 52% lower (*P* < 0.05); however, there was no difference in the respiratory control ratio (RCR) (Fig. 1*B*). Moreover, OXPHOS respiration supported by succinate as a substrate for the S-pathway via complex II (S_*P*_) was decreased by 32% (*P* = 0.082) between PE < 34 wk placentas and NTC.

Next, ETC complexes were evaluated at the molecular level. Muralimanoharan et al. (27) reported that subunits of ETC complexes were reduced in placentas from late-onset (∼38 wk) PE. As NTC and PE < 34 wk placentas showed significant differences in gestational age and placental mitochondrial content may alter as pregnancy progresses, NPTCs were also evaluated (Table 2). These were considered valid controls as it is unlikely that protein levels of the complexes change significantly during the duration of labor. ETC complexes were assayed using an antibody mixture to quantify representative subunits by Western blot. The subunits detected were NDUFB8 (complex I), SDHB (complex II), UQCRC2 (complex III), MT-CO1 (complex IV), and ATP5F1A (ATP synthase), since these subunits are labile when the complex is not assembled. Levels of all complex subunits were unaltered in PE < 34 wk placentas, with the exception of UQCRC2, which was ∼50% lower in PE placentas compared with NTC (*P* < 0.05), though not different from NPTC (Fig. 1 *C* and *D*). Citrate synthase was used to normalize ETC subunits as it is a putative biomarker of mitochondrial content (31, 32). There were no differences in citrate synthase (CS) among NPTC, NTC, and PE < 34 wk placentas; however, its level showed greater variability in PE placentas (Fig. 1 *C* and *D*). RNA sequencing (RNA-seq) was used to investigate the expression of all 97 ETC complex subunit genes. Only a small number of genes showed significant variation by ∼20% compared to NTC (*SI Appendix*, Fig. S1).

**Table 2.** Clinical characteristics of placentas for Western blotting analysis

Characteristics	NPTC (*n* = 7)	NTC (*n* = 7)	PE < 34 wk (*n* = 7)	*P* value	
				NPTC vs. PE	NTC vs. PE
Gestational age, wk	29.4 ± 3.3	39.3 ± 0.4	30.3 ± 1.1	ns	*P* < 0.001
Systolic blood pressure	115.9 ± 9.9	124 ± 7.9	163 ± 13.9	*P* < 0.001	*P* < 0.001
Diastolic blood pressure	75.1 ± 10.2	72 ± 10.6	101.9 ± 4.7	*P* < 0.001	*P* < 0.001
Birth weight, g	1,373 ± 690	3,680 ± 392	991 ± 80	ns	*P* < 0.001
Placental weight, g	249 ± 67	566 ± 173	171 ± 30	*P* < 0.05	*P* < 0.001

ns, not significant.

### rHR Recapitulates OXPHOS Capacity Changes Observed in the PE < 34 wk Placentas.

Placental oxidative stress induced by ischemia–reperfusion resulting from insufficient spiral arteries remodelling is thought to be central to the pathophysiology of early-onset PE (6). By fluctuating the oxygen concentration between 1% (hypoxia) and 20% (reoxygenation) in 6-h cycles (rHR), we were able to activate the UPR^ER^ in trophoblast-like cells to a severity similar to that observed in the placenta in PE < 34 (4). In the present study, the same model was used to investigate whether rHR could induce equivalent changes related to the mitochondrial dysfunction as observed in vivo, and to explore the molecular mechanisms suppressing mitochondrial activity in BeWo cells.

**Fig. 2.**
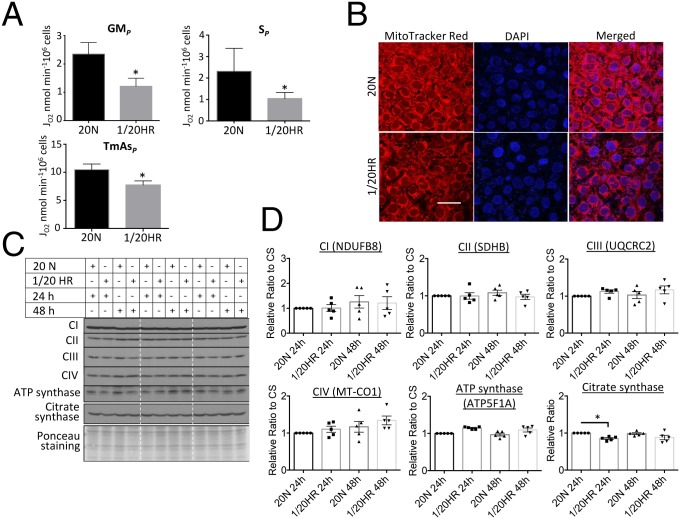
rHR recapitulates the mitochondrial changes observed in the PE < 34 wk placenta. BeWo cells were subjected to rHR for 48 h. (*A*) rHR reduces OXPHOS capacity supported by substrates for N-pathway via complex I (GM_*P*_), S-pathway via complex II (S_*P*_), and nonphysiological electron donors to complex IV (TmAs_*P*_). After addition of substrates, rate of oxygen consumption of cells was measured and data are presented as mean ± SEM, *n* = 4, *P < 0.05 (2-tailed paired *t* test). (*B*) rHR reduces mitochondrial membrane potential. Cells were stained with MitoTracker Red before being fixed, permeabilized, and stained with nuclear dye DAPI. Images were taken with confocal microscopy with 400× magnification. (Scale bar: 50 μm.) (*C* and *D*) Expression of ETC complexes subunits does not alter under rHR. The level of 5 ETC subunits was quantified using OXPHOS antibody mixture. Data were normalized to CS before expressing as a relative ratio to normoxic control, which was set as 1. Data are presented as mean ± SEM, *n* = 5. No significant change of all ETC complexes subunits (1-way ANOVA with Holm–Sidak's multiple comparisons test). The 20 N indicates cells were incubated under normoxic conditions with 20% O_2_ for 24 or 48 h; 1/20 HR indicates cells were exposed to a 6-h cyclic pattern of 1% and 20% O_2_ for 24 or 48 h.

Mitochondrial respiration was examined in rHR-treated cells. OXPHOS capacity was 48% lower (*P* < 0.05) with malate and glutamate as substrates (N-pathway through complex I, GM_*P*_), and 55% lower (*P <* 0.05) with succinate as a substrate (S-pathway through complex II, S_*P*_) (Fig. 2*A*). In addition, rHR-treated cells showed a 26% (*P* < 0.05) reduction in complex IV-supported respiration (TmAs_*P*_) (Fig. 2*A*). Loss of OXPHOS capacity was associated with a reduction in mitochondrial membrane potential as indicated by MitoTracker Red fluorescence (Fig. 2*B*). We then investigated compromise of ETC subunit proteins using the OXPHOS antibody mixture. None of the 5 representative subunits showed down-regulation (Fig. 2 *C* and *D*), and the level of CS remained constant (Fig. 2 *C* and *D*). RNA-seq was used to assess the expression of all 97 ETC subunit genes in the rHR-treated BeWo cells, with most genes showing 5 to 30% variation in expression (*SI Appendix*, Fig. S2). Expression of *SDHB* and *UQCRC2* decreased by 39% and 22% (*P* < 0.001), respectively, despite the fact that protein levels remained constant (Fig. 2*D* and *SI Appendix*, Fig. S2).

### Activation of Noncanonical UPR^mt^ in rHR-Treated Cells.

**Fig. 3.**
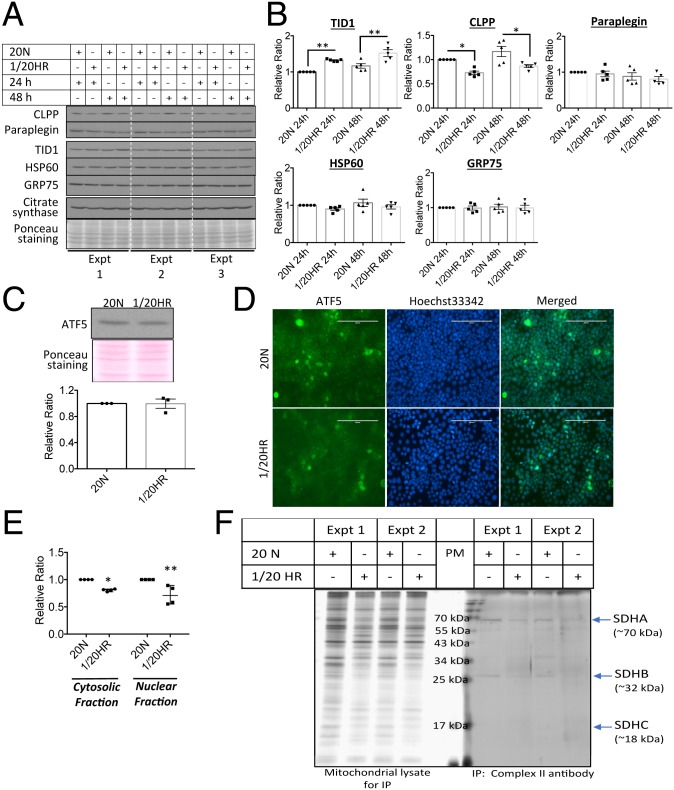
rHR activates a noncanonical UPR^mt^ pathways. (*A* and *B*) rHR triggers noncanonical UPR^mt^ pathways. BeWo cells were subjected to rHR for 24 and 48 h. Western blot was used for measurement expression of UPR^mt^ molecular markers CLPP, paraplegin, TID1, HSP60, GRP75, and CS. Data were normalized to CS and are expressed as mean ± SEM, *n* = 5. *P < 0.05; **P < 0.01 (2-tailed paired *t* test at either 24 h or 48 h). (*C*–*E*) No increase in cellular expression but decreased nuclear translocation of ATF5 under rHR. Cells were exposed to 48 h of rHR. Western blot was used to quantify ATF5 while immunocytochemistry and subcellular fractionation was used to show its cellular localization. Data are presented as mean ± SEM, *n* = 3 to 4. *P < 0.05; **P < 0.01 (2-way ANOVA with Sidak’s multiple comparisons test). Magnification, 200×. (Scale bar: 200 μm.) (*F*) Potential conformation change of ETC complexes. Isolated mitochondria were subjected to immunoprecipitation with conformation-sensitive mitoprofile complex II antibody to pull out complex II before resolving in SDS/PAGE gel. Silver staining was used to reveal 4 subunits of complex II. The 20 N indicates cells were incubated under normoxic conditions with 20% O_2_ for 24 or 48 h; 1/20 HR indicates cells were exposed to a 6-h cyclic pattern of 1% and 20% O_2_ for 24 or 48 h.

Several regulatory components link the UPR with mitochondrial regulation and function (13). Therefore, the activation of UPR^mt^ in the rHR-treated cells was investigated. rHR treatment up-regulated and down-regulated the UPR^mt^ biomarkers TID1 and CLPP by 33% (*P* < 0.01) and 27% (*P* < 0.01), respectively, after 24 h (Fig. 3 *A* and *B*). Prolonged challenge up to 48 h caused no further change (Fig. 3 *A* and *B*). Other UPR^mt^ biomarkers, HSP60, GRP75 and paraplegin, remained constant throughout the 48-h challenge. There was only a minimal degree of cell death (<1%) observed after 48 h. We observed a subtle reduction of CS by 15% at 24 h, but not at 48 h (Fig. 3*B*). The transcription factor ATF5 regulates mammalian UPR^mt^ gene expression, including *HSP60*, *GRP75*, and *CLPP* (24). However, neither changes in the cellular level nor nuclear localization of ATF5 were observed in rHR-treated cells (Fig. 3 *C* and *D*), but subcellular fractionation indicated a significant down-regulation of both cytosolic and nuclear ATF5 by 19% (*P* < 0.05) and 28% (*P* < 0.01), respectively (Fig. 3*E* and *SI Appendix*, Fig. S3), indicating the presence of ATF5 in other cellular organelles.

Activation of the UPR^mt^ indicates potential accumulation of unfolded/misfolded proteins in the mitochondrial matrix. However, it is technically challenging to detect misfolded subunits of the ETC complexes directly. Therefore, an indirect approach based on immunoprecipitation was adopted, in which a conformation-sensitive antibody was used to pull down the target protein. We selected complex II as the target protein as it contains only 4 subunits (SDHA, SDHB, SDHC, and SDHD). An anti-complex II Mitoprofile antibody was used for immunoprecipitation in isolated mitochondrial lysates of BeWo cells. Less SDHB was detected in the immunoprecipitated complex II (Fig. 3*F*), while no change was observed in the denatured gel (Fig. 2*D*), suggesting possible misfolding of ETC subunits. Further studies will be required to confirm this finding.

### UPR^mt^ Suppresses Mitochondrial Oxidative OXPHOS Activity.

**Fig. 4.**
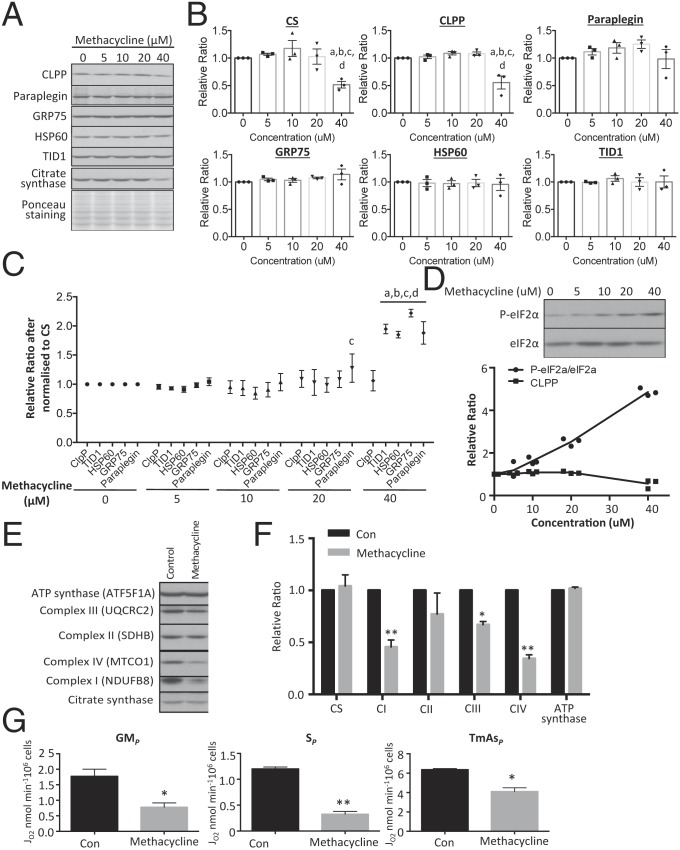
Activation of UPR^mt^ impairs mitochondrial OXPHOS capacity. Cells were treated with the UPR^mt^ inducer methacycline for 24 h or 72 h. (*A*–*C*) Methacycline suppresses levels of mitochondrial CS and CLPP proteases and ETC complex subunits but not chaperones and ATP synthase in a dose-dependent manner. (*A*) Expression of CLPP, paraplegin, TID1, HSP60, GRP75, and CS were measured by Western blot. (*B*) Band intensity of mitochondrial chaperones and OXPHOS complexes subunits was quantified before expressing as a relative ratio to untreated control, which was set as 1. (*C*) Data were normalized to CS before expressing as a relative ratio to untreated control, which was set as 1. In *B* and *C*, data are presented as mean ± SEM, *n* = 3, and were analyzed using a 2-way ANOVA with Tukey’s multiple comparison test. a, b, c, and d indicate statistically significant changes at methacycline concentrations of 0, 5, 10, or 20 μM, respectively. (*D*) Methacycline promotes phosphorylation of eIF2α. There was a dose-dependent increase of phosphorylation with increasing concentration of methacycline. The increase P-eIF2α is closely associated with the decrease of CLPP protein. Band intensity of P-eIF2α and eIF2α was quantified and the ratio between phosphorylated and total was calculated before expressing as a relative ratio to untreated control, which was set as 1. (*E* and *F*) Prolonged treatment wth methacycline inhibits expression of ETC complex subunits selectively. Cells were incubated with sublethal dosage of methacycline (20 μM) for 72 h. Data are expressed as relative ratio to the untreated control, which was set as 1, and are presented as mean ± SEM, *n* = 3. Ponceau S staining was used to show equal loading in Western blot. (*G*) Methacycline reduces OXPHOS capacity supported by substrates for N-pathway via complex I (GM_*P*_), S-pathway via complex II (S_*P*_), and nonphysiological electron donors to complex IV (TmAs_*P*_). Data are presented as mean ± SEM, *n* = 4, as the amount of oxygen being consumed by 10^6^ of cells per min. For *F* and *G*, *P* < 0.05 is considered statistically significant. *P < 0.05; **P < 0.01 under 2-tailed paired Student’s *t* test.

Next, we investigated whether activation of the UPR^mt^ is sufficient to modulate OXPHOS capacity. A number of agents capable of inducing the UPR^mt^ have been identified through a screen in *C. elegans* (33). However, their efficacy in mammalian cells is unknown. Therefore, we chose the most promising, methacycline, and tested it on BeWo cells. A dose–response study of methacycline up to 40 μM for 24 h revealed that the chaperones HSP60, GRP75, and TID1 and paraplegin did not change, while the protease CLPP reduced significantly (*P* < 0.01) after 40 μM. However, CS also showed down-regulation by 50% (*P* < 0.01) at 40 μM (Fig. 4 *A* and *B*). After normalization to CS, levels of TID1, HSP60, GRP75 and paraplegin (*P* < 0.01) were increased after 40 μM of methacycline, whereas CLPP was unchanged (Fig. 4*C*). These results confirm that methacycline can induce UPR^mt^ in BeWo cells and low CS protein level may implicate loss of mitochondrial content. Interestingly, methacycline treatment also induced a dose-dependent activation of phosphorylation of eIF2α and was closely correlated with reduction of CLPP protein (Fig. 4*D*).

To study the effect of UPR^mt^ on mitochondrial activity, BeWo cells were incubated at a lower concentration of methacycline (20 μM) for a longer period of 72 h. In this case, methacycline did not affect CS (Fig. 4 *E* and *F*). However, the treatment did suppress levels of NDUFB8, UQCRC2, and MT-CO1 by 44%, 33%, and 66% (*P* < 0.01, *P* < 0.05, and *P* < 0.01), respectively (Fig. 4 *E* and *F*). OXPHOS capacity was measured in methacycline-treated cells after 72-h incubation and complex I (GM_*P*_)-, complex II (S_*P*_)-, and complex IV (TmAs_*P*_)-supported OXPHOS were reduced by 56% (*P* < 0.05), 73% (*P* < 0.01) and 36% (*P* < 0.05), respectively (Fig. 4*G*). A comparison between OXPHOS capacities (Fig. 4*G*) and their corresponding complex subunit protein levels (Fig. 4*F*) revealed that the decreased mitochondrial respiration with substrates for the N-pathway via complex I and electron donors for complex IV could be accounted for by the reduction of protein levels. For complex II, there was no significant change in protein level while mitochondrial respiration via the S-pathway through complex II decreased over 70%, (*P* < 0.01), suggesting that another mechanism may be involved. This may be due to incorrect quaternary structure, associated with the UPR^mt^, or alternatively electron flow along the S-pathway might be impaired downstream at complex IV, possibly related to altered mitochondrial supercomplex assembly (34).

### Down-Regulation of *CLPP* Is Sufficient to Compromise Mitochondrial Function.

**Fig. 5.**
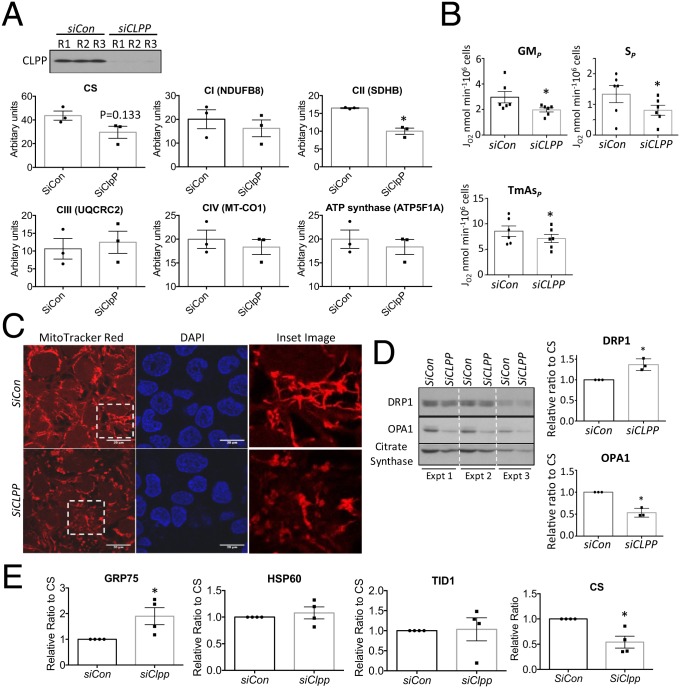
Knockdown of CLPP gene suppresses complex II expression, inhibits OXPHOS capacity, and promotes mitochondrial fission. *CLPP* was knocked down by small RNA interference either for 48 h (*A*) or subsequent subculturing for additional 72 h (*B*–*E*) prior to experimentation. (*A*) Short-term down-regulation of CLPP reduces complex II (SDHB) expression. Western blot was used to measure ETC complexes subunits with OXPHOS antibody mixture. Data were normalized to CS and are presented as mean ± SEM, *n* = 3. (*B*) Long-term suppression of CLPP protein reduces activity of complex II. Respirometry was used to measure oxygen consumption in both *SiCon* and *SiCLPP*-transfected cells. Data were normalized to cell density and expressed as mean ± SEM, *n* = 6. *P < 0.05. (*C*) Loss of CLPP protein diminishes mitochondrial membrane potential and promotes fragmentation. Mitochondrial membrane potential was measured by MitoTracker Red in cells prior to fixation, and nuclei were counterstained with DAPI. Images were taken under confocal microscope. (Scale bars: 20 μm.) Insets are digital zoom-in images. (*D*) Reduction of CLPP facilitates mitochondrial fission. Western blot was used to quantify expression of mitochondrial fission and fusion markers DRP1 and OPA1, respectively. Data are presented as mean ± SEM, *n* = 3. *P < 0.05. (*E*) Chronic loss of CLPP decreases mitochondrial density and promotes UPR^mt^. Western blotting was used to measure CS and UPR^mt^ biomarkers. Data were normalized to CS and are presented as mean ± SEM, *n* = 4. *P < 0.05. All data were analyzed by 2-tailed paired Student’s *t* test.

To investigate whether reduction of CLPP affects OXPHOS capacity, induces mitochondrial dysfunction, and activates UPR^mt^, small interfering RNA (siRNA) was used to knock down *CLPP* in BeWo cells. There was >95% reduction of CLPP protein after 48 h of transfection (Fig. 5*A*). Interestingly, short-term down-regulation of CLPP affected complex II, with expression of the subunit SDHB reduced by over 35% (*P* < 0.05), while the other 4 complexes subunits detected by the OXPHOS antibody mixture remained unchanged (Fig. 5*A*). CS also showed a reduction in the *SiCLPP*-transfected cells, but the change did not reach statistical significance (Fig. 5*A*). There was no change in mitochondrial membrane potential or morphology (*SI Appendix*, Fig. S4*A*). In order to investigate the long-term consequence of *CLPP* knockdown on mitochondrial function, after 48 h of transfection *SiCLPP*-transfected cells were subcultured for an additional 72 h. CLPP protein was persistently suppressed by over 95% throughout (*SI Appendix*, Fig. S4*B*). Respirometry analysis revealed that both complex I- (GM_*P*_), II- (S_*P*_), and IV- (TmAs_*P*_) supported OXPHOS were reduced by 20 to 50% (*P* < 0.05) (Fig. 5*B*).

MitoTracker Red fluorescence showed strong staining in the elongated tubular network of mitochondria in control (*SiCon*) cells, while knockdown of *CLPP* produced a fragmented pattern with weaker staining except for few “hot spots” which had high membrane potential, indicating partial loss of mitochondrial membrane potential (Fig. 5*C*). Serial confocal microscopy images eliminated a potential artifact arising from the orientation of the mitochondria (*SI Appendix*, Fig. S4*C*). Mitochondria undergo dynamic fusion or fission in response to stimuli or stress. Indeed, there was a 37% (*P* < 0.05) increase in the fission protein, DRP1, and a 47% (*P < 0.05*) decrease of the fusion protein, OPA1, after normalization to CS in the *SiCLPP*-transfected cells (Fig. 5*D*). Long-term suppression of *CLPP* expression reduced CS (*P* < 0.05). After normalization to CS, GRP75 was found to be up-regulated (*P* < 0.05) (Fig. 5*E*), suggesting potential accumulation of misfolded proteins and activation of UPR^mt^. These results indicate a dynamic change of mitochondrial function, morphology, and density in response to low CLPP.

### UPR^ER^ Regulates CLPP Expression through eIF2α at Both Transcriptional and Translational Levels in an ATF4-Independent Pathway.

**Fig. 6.**
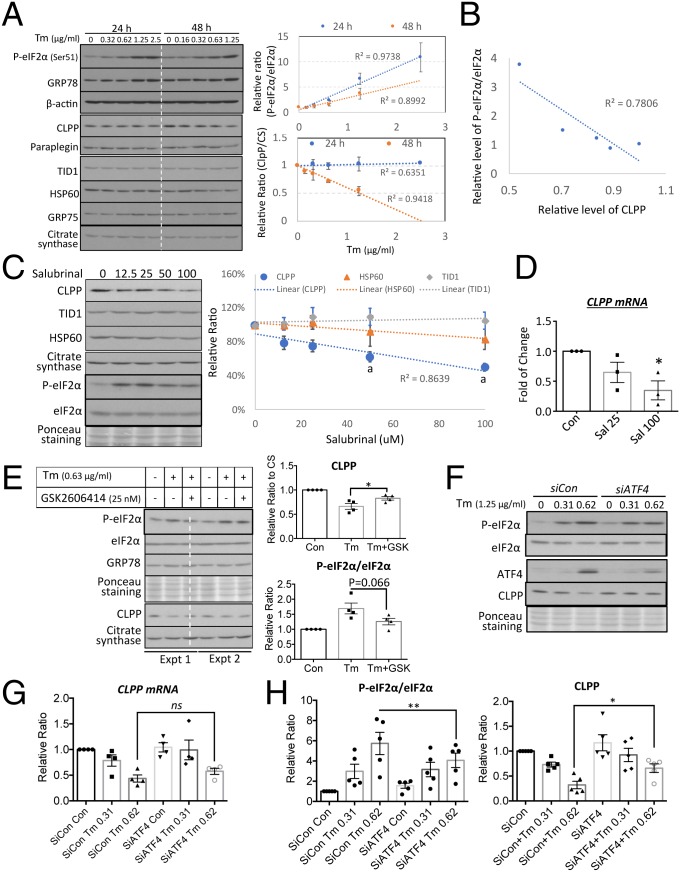
Prolonged rather than acute UPR^ER^ suppresses CLPP expression in a severity-dependent manner through a PERK/eIF2α but ATF4-independent pathway. Tunicamycin was used to activate UPR^ER^ for 24 or 48 h. (*A*) Prolonged UPR^ER^ suppresses CLPP in the absence of change of other UPR^mt^ markers. Cells were treated with tunicamycin ranging from 0.31 to 2.5 μg/mL for 24 h or 0.16 to 1.25 μg/mL for 48 h. Levels of CLPP were normalized to CS. The relative levels of P-eIF2α/eIF2α and CLPP were plotted against concentrations of tunicamycin at both 24 and 48 h and a linear regression line was fitted. (*B*) A strong correlation between P-eIF2α/eIF2α ratio and CLPP. Scatter plot was constructed between P-eIF2α/eIF2α and CLPP and a linear regression line was fitted. (*C*) Phosphorylated eIF2α suppresses CLPP. Cells were subjected to a dose–response treatment with salubrinal for 24 h. The levels of CLPP, TID1, and HSP60 were normalized to CS before plotting against the concentration of salubrinal. ^a^*P* < 0.05 compared to untreated control. (*D*) Down-regulation of CLPP by salubrinal is at the transcriptional level. qRT-PCR was used to measure *CLPP* transcripts. Data are presented as mean ± SEM, *n* = 3. *P < 0.05. (*E*) Inhibition of eIF2α phosphorylation restores CLPP. Cells were treated with tunicamycin (0.63 μg/mL) with or without the PERK-specific inhibitor GSK2606414 for 48 h. Data are presented as mean ± SEM, *n* = 4. *P < 0.05. (*F* and *G*) ER stress-mediated down-regulation of CLPP is independent of ATF4. qRT-PCR was used to measure *CLPP* transcripts. Data are presented as mean ± SEM, *n* = 4. (*H*) Phosphorylation status of eIF2α regulates CLPP translation. Knockdown of ATF4 reduced phosphorylation eIF2α and was accompanied by an increase of CLPP in the absence of *CLPP* transcript change. Data are presented as mean ± SEM, *n* = 5, *P < 0.05; **P < 0.01. Statistical analysis was performed using a 2-tailed paired Student’s *t* test.

Increasing evidence demonstrates an interplay between the UPR^ER^ and UPR^mt^ (13, 22). Coincidently, activation of UPR^mt^ by methacycline is associated with increased P-eIF2α and reduced CLPP. Therefore, we investigated the relationship between the UPR^ER^ in down-regulation of CLPP in trophoblast-like cells. Tunicamycin inhibits initiation of N-linked glycosylation in the ER lumen and is a widely used and highly specific UPR^ER^ inducer with minimal direct effects on mitochondria. The drug was used in BeWo cells to perform both dose–response and time-course analyses. The UPR^ER^ biomarkers P-eIF2α and GRP78 increased gradually with rising concentrations of tunicamycin (Fig. 6 *A*, *Upper Right*). There was no change in CLPP after 24 h, but after 48 h of incubation there was a strong negative correlation (*R*^2^ = 0.9418) between the level and the concentration of tunicamycin (Fig. 6 *A*, *Lower Right*). Other UPR^mt^ biomarkers were unchanged. Indeed, there was a strong correlation (*R*^2^ = 0.7806) between the P-eIF2α/eIF2α ratio and CLPP levels (Fig. 6*B*). Therefore, we investigated the potential role of the PERK/eIF2α/ATF4 pathway in regulation of *CLPP* expression and/or protein level. Salubrinal inhibits dephosphorylation of eIF2α and causes elevation of P-eIF2α in the absence of the UPR^ER^ (35). Administration of salubrinal led to a dose-dependent down-regulation of CLPP without any change in TID1, HSP60, and CS after 48 h (Fig. 6*C*). Next, we investigated whether the effect is mediated by transcriptional or translational regulation. *CLPP* mRNA was also reduced under salubrinal as measured by qRT-PCR, indicating transcriptional regulation (Fig. 6*D*). To further confirm the role of eIF2α in suppression of CLPP expression, we inhibited the upstream kinase of eIF2α, PERK, with the specific inhibitor GSK2606414 (36). Results presented in Fig. 6*E* showed that application of GSK2606414 inhibited phosphorylation of eIF2α induced by tunicamycin and partially restored CLPP protein level (*P* < 0.05).

ATF4 and CHOP are transcription factors and downstream effectors of the PERK/eIF2α pathway. CHOP has been shown to up-regulate, rather than suppress, *CLPP* expression (37). Therefore, we investigated the role of ATF4 in suppression of *CLPP* expression using siRNA. *SiATF4* transfection greatly suppressed the increase in ATF4 in response to tunicamycin but failed to restore *CLPP* transcript levels (Fig. 6 *F* and *G*). Suppression of ATF4 decreased levels of phosphorylated eIF2α by ∼30% (*P* < 0.01) and was accompanied by a 1.1-fold increase of CLPP protein (*P* < 0.05), despite no change in *CLPP* mRNA in *SiATF4*-transfected cells in the presence of tunicamycin (Fig. 6*H*). These results indicate ATF4 is not involved in the negative regulation of *CLPP* expression but do suggest translational regulation of CLPP by the phosphorylation status of eIF2α.

### Similar Activation of Noncanonical UPR^mt^ Pathway in PE < 34 wk Placentas.

**Fig. 7.**
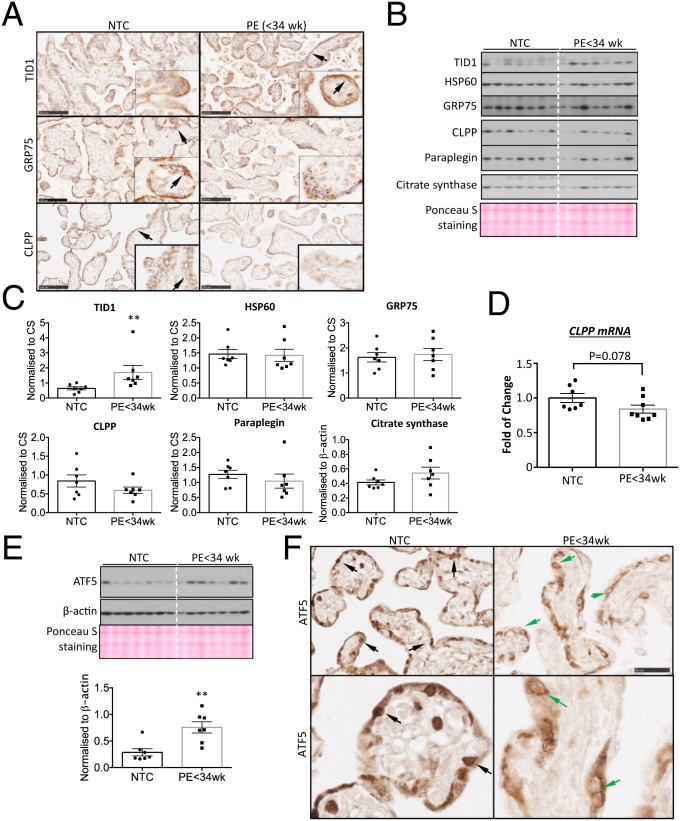
Existence of noncanonical UPR^mt^ pathway in the PE < 34 wk placentas. (*A*) UPR^mt^ biomarkers TID1, GRP75, and CLPP are localized mainly in the syncytiotrophoblast (arrows) and there is a down-regulation and up-regulation of CLPP and TID1, respectively, in the PE < 34 wk placentas. (Scale bars: 100 μm.) (*B* and *C*) Low-grade activation of UPR^mt^ is detected in the PE < 34 wk placentas. Expression of 5 UPR^mt^ markers was examined by Western blot. Ponceau S staining was used as a loading control. Band intensities were quantified and normalized to CS and are presented as mean ± SEM, *n* = 7. **P < 0.01. (*D*) *CLPP* transcript is reduced in the PE < 34 wk placentas. Quantitative real-time RT-PCR was used to measure *CLPP* transcript level. Data are presented as mean ± SEM, *n* = 7. (*E*) Elevation of ATF5 expression in the PE < 34 wk placentas. Expression of ATF5 protein was quantified by Western blot. Both β-actin and Ponceau S staining were used as loading controls. Band intensities were quantified and normalized to β-actin and are presented as mean ± SEM, *n* = 7. **P < 0.01. (*F*) ATF5 does not translocate into nuclei of the PE < 34 wk placentas. Immunohistochemical staining was used to show cellular localization of ATF5. (Upper panel is at 100× magnification, scale bar: 100 μm; lower panel is at 200× magnification, scale bar: 50 μm.) Inset images show nuclear staining of ATF5 in the NTC (black arrows) but perinuclear staining (green arrows) in the PE < 34 wk placentas. All data were analyzed by 2-tailed unpaired Student’s *t* test.

Finally, we examined evidence of activation of this noncanonical UPR^mt^ pathway, which precipitates mitochondrial dysfunction, in PE < 34 wk placentas. Activation of the placental UPR^ER^ in early-onset PE is principally immunolocalized to the syncytiotrophoblast and endothelial cells (38). Therefore, immunohistochemistry was first used to identify the cell types that display activation of the UPR^mt^. Indeed, the UPR^mt^ was largely restricted to the syncytiotrophoblast (Fig. 7*A*). Punctate staining for TID1, GRP75, and CLPP was observed, typical of mitochondrial localization (Fig. 7*A*). In PE < 34 wk placentas, the staining for TID1 and GRP75 was increased and unchanged, respectively. CLPP staining was less intense in the syncytiotrophoblast (Fig. 7 *A*, *Bottom*) in the PE < 34 wk placentas.

Western blotting was then used to quantitate expression of UPR^mt^ biomarkers. NPTC placentas were excluded as the labor process strongly activates UPR^ER^ pathways (30). Indeed, the UPR^mt^ biomarkers were also increased in those placentas, although the differences did not reach statistical significance (*SI Appendix*, Fig. S5). The cochaperone TID1 showed a 2-fold (*P* < 0.01) increase in PE < 34wk placentas compared to NTC (Fig. 7 *B* and *C*). Additionally, the 2 key mitochondrial chaperones, HSP60 and GRP75, were unchanged, while the quality-control proteases CLPP and paraplegin showed trends toward a decrease (Fig. 7 *B* and *C*). CLPP was further quantified at the gene level by qRT-PCR and showed a trend toward a ∼20% decrease (*P* = 0.078) in PE < 34 wk placentas (Fig. 7*D*). When considering the significance of these results, it must be remembered that the syncytiotrophoblast where the UPR^mt^ biomarkers are almost exclusively expressed represents only ∼30% of villous volume. Consequently, changes in this cell type will not necessarily be accurately reflected in villous lysates.

Despite no change in ATF5 in rHR-treated cells, in PE < 34 wk placentas we observed a 2.6-fold increase in ATF5 protein (*P* < 0.01; Fig. 7*E*). However, immunostaining of ATF5 was mainly observed in the syncytioplasm and nuclear localization was largely absent (Fig. 7*F*). Additionally, there was some perinuclear staining of ATF5 in the cytotrophoblast cells, identified by their large nuclear features (Fig. 7*F*). Failure of ATF5 to translocate into the nucleus may explain the lack of increase in its target genes *HSP60* and *GRP75* under mitochondrial stress. To conclude, these results are very similar to the rHR-treated BeWo cells.

## Discussion

Mitochondrial dysfunction has been widely reported in placentas of complicated pregnancies, including PE (39), and may contribute to the pathophysiology. In this study, we first demonstrated a reduction of mitochondrial respiration in situ and showed that this could be recapitulated by exposure of trophoblast-like cells to rHR challenge. We then elucidated activation of a noncanonical UPR^mt^ pathway in the challenged cells. The key mitochondrial quality-control protease, CLPP, was down-regulated, while the cochaperone TID1 was elevated. We demonstrated that activation of the UPR^mt^ pathway in vitro is sufficient to modulate mitochondrial respiration, and that depletion of CLPP inhibits OXPHOS capacity and facilitates mitochondrial fission. We also identified translational regulation of CLPP by the PERK/eIF2α signaling independent of ATF4 in the UPR^ER^ pathway. Finally, we observed evidence of activation of the same pathways in placental samples from early-onset PE. We have previously demonstrated that UPR^ER^ pathways are not activated in placentas from late-onset PE (4) above the levels seen in NTCs. Indeed, there is also no change in UPR^mt^ markers (*SI Appendix*, Fig. S6).

In the current literature, the majority of studies in PE have focused on changes in placental mitochondrial content, and results have been inconsistent (39). This may be due to the difficulty in assessing content, as there are no reliable markers of mitochondrial number other than ultrastructural analysis (32). Indeed, functional approaches, which were used in the present study, are more appropriate. Only a few studies have attempted to investigate the mechanisms underlying mitochondrial dysfunction using either isolated primary trophoblast cells or mitochondria isolated from preeclamptic placentas (27, 28). These are not ideal models as the data do not reflect the activity of mitochondria in situ. A recent study measured mitochondrial respiration in late-onset preeclamptic placental tissues in situ (40). Unfortunately, the placental samples were obtained from a mixture of caesarean and vaginal deliveries, making it difficult to interpret the results for labor-induced oxidative damage can compromise mitochondrial function (41). In our study, all term control and preeclamptic placental tissues were obtained from nonlabored caesarean deliveries.

Ischemia–reperfusion resulting from insufficient remodelling of the uterine spiral arteries has been proposed as the major trigger of placental oxidative and ER stress in early-onset PE (4, 6). Here, we were able to recapitulate the in vivo molecular changes in mitochondrial function in the PE < 34 wk placentas with the rHR model of trophoblast-like BeWo cells cultured under 5.5 mM glucose (4). Culturing cells at 5.5 mM physiological glucose level is crucial in rHR to avoid metabolic acidosis due to the high glucose concentrations in routine commercially available culture media (42). This rHR model may therefore provide a useful tool for studying the placental stress occurring in early-onset PE.

The mechanism by which the UPR^mt^ suppresses mitochondrial respiration in rHR is unclear. However, down-regulation of the quality-control protease CLPP, which is involved in proteostasis, and high expression of cochaperone TID1 in rHR-treated cells provide possible explanations. Knockdown of *CLPP* decreases ETC complex II SDHB expression and also inhibits OXPHOS capacity (Fig. 6). These results are consistent with the study by Cole et al. (43) in which genetic knockdown of *CLPP* impaired ETC complex II and OXPHOS in OCI-AML2 cells. On the other hand, TID1 can interact directly with ETC complex I and suppress its activity (44). These findings illustrate the direct regulatory role of UPR^mt^ pathway in mitochondrial OXPHOS capacity and function. Interestingly, we found that knockdown of *TID1* suppressed, while knockdown of the protease *CLPP* stimulated, ER stress induced by tunicamycin (*SI Appendix*, Fig. S4*D*). These findings suggest that low CLPP and high TID1 levels may form a feedback loop between the UPR^mt^ and UPR^ER^. Therefore, the role of up-regulation of TID1 in mitochondrial function in the rHR-treated cells deserves further investigation.

Activation of the UPR^mt^ in our samples appeared to be associated with mitochondrial fragmentation or fission, and presumed removal by mitophagy. These changes may reflect attempts to recycle damaged organelles (45, 46). Data on placentas from PE are contradictory, since both an increase (28) and decrease in the fusion protein OPA1 (47) have been reported. This variation may reflect differences in the degree and duration of the stress in the respective patient groups, for low stress levels increase fusion and/or decrease fission, whereas high stress levels stimulate the opposite (48).

We did not include the *clpp*^*−/−*^ transgenic mouse model in this study because of the major structural differences between the rodent and human placenta that make it impossible to interpret equivalent UPR^mt^ findings in the mouse. In human placenta, the syncytiotrophoblast of the placental villi performs both endocrine and nutrient exchange functions. By contrast, the mouse placenta is composed of 2 highly specialized regions, the junctional zone for endocrine activity and the labyrinth zone for nutrient exchange. Due to its high demand of energy for active transport of nutrients, the labyrinth zone has the highest mitochondrial activity (49). Therefore, this zone is susceptible to the UPR^mt^ but is relatively insensitive to the UPR^ER^ due to the low density of ER (50). Conversely, the UPR^ER^ exists only in the junctional zone that has a high synthetic and secretory activity of peptide hormones (50). In the preeclamptic placenta, the UPR^mt^ is localized exclusively to the syncytiotrophoblast, where it colocalizes with the UPR^ER^ (38). This allows the PERK-eIF2α of UPR^ER^ pathway to negatively regulate CLPP gene and protein expression. Therefore, the phenomenon of a noncanonical UPR^mt^ pathway in human placenta is unlikely to be replicated in the mouse because UPR^ER^ and UPR^mt^ are selectively activated in 2 different regions and cell types.

Furthermore, in the *siCLPP*-treated BeWo cells, we observed down-regulation of complex I, III, and IV subunits. A study by Szczepanowska et al. (51) showed that in the *clpp*^*−/−*^ mouse the effect of CLPP on expression of mitochondrial ETC subunits appears to be closely related to the respiratory activity of the cells concerned. Thus, they observed no change of ETC complex subunits as recognized by the OXPHOS antibody mixture in the liver, a decrease of complex IV subunit in the heart, and a reduction of both complex III and IV subunits in skeletal muscles. Therefore, the higher the mitochondrial activity, the stronger the effect of CLPP in suppressing mitochondrial protein expression. Indeed, loss of CLPP decreases formation of mitoribosomes, thereby diminishing mitochondrial protein synthesis (51). Hence, the effect of *CLPP* knockdown on ETC complexes may be different in the human and murine placenta. Nonetheless, *clpp*^*−/−*^ mice show a partially embryonic lethality and surviving pups exhibit growth retardation and die prematurely in adulthood, indicative of placental insufficiency (52).

In *CLPP* knockdown cells, mitochondrial fragmentation was only observed 5 d after transfection (Fig. 5*D* and *SI Appendix*, Fig. S4*A*). Some of the fragmented mitochondria still maintained a high membrane potential. Interestingly, a recent study suggested that mitochondrial stress-mediated ATP depletion facilitates generation of “hot spots” across the mitochondrial network (44). Within those “hot spots” the mitochondrial membrane potential is maintained, thereby partially restoring OXPHOS capacity and maintaining ATP production. The unique structure of the syncytiotrophoblast, with no lateral cell boundaries, will permit free movement of organelles and metabolites between “hot spots” and areas where mitochondrial function is impaired. Hence, integrity and function may be preserved to a greater degree than in unicellular tissues.

The UPR^ER^ and UPR^mt^ pathways are closely linked (13), and activating one pathway is likely to trigger the other. Our results revealed that the duration of the ER stress insult, rather than its severity, is a key component in activation of the UPR^mt^ pathway. There may be additional interactions between the ER and mitochondria, as ATF4, a downstream transcription factor in the PERK arm of the UPR^ER^ pathway controls expression of the ubiquitin ligase Parkin, a key regulator of mitochondrial function and dynamics (53). The transcription factor CHOP, the other PERK downstream effector, also positively regulates expression of key genes in the UPR^mt^ pathway, including *HSP60*, *TID1*, and *CLPP* (23). In this study, we also revealed the potential role of eIF2α in both transcriptional and translational regulation of *CLPP* expression and protein synthesis in an ATF4-independent pathway. Genes with a universal open reading frame (uORF) or an internal ribosome entry site sequence in their promoter can bypass eIF2α regulation (54, 55). No uORFs were found in the promoter region of *CLPP* gene up to −123 bp of the 5′ untranslated region, suggesting potential translation attenuation upon phosphorylation of eIF2α. Upon severe stress with irreversible cellular damage, this mechanism provides a negative feedback that attenuates the UPR^mt^ protective pathway, thereby activating mitochondrial-mediated apoptosis to eliminate the cells.

ATF5 was increased in the placental samples from PE but not in the rHR-treated cells. The reason(s) behind this difference is unclear. ATF5 has been shown to be regulated by CHOP (56). However, although CHOP was found to localize in the nuclei of the rHR-treated cells (*SI Appendix*, Fig. S7) and the syncytiotrophoblast of the placenta from early-onset preeclampsia (14), ATF5 was only increased in the placenta, suggesting another inhibitory mechanism likely interacts with CHOP in regulating ATF5 expression under hypoxia–reoxygenation. Nonetheless, neither case had nuclear localization of ATF5, which may explain why there was no increase in the folding and quality control chaperones HSP60 and GRP75, typical biomarkers for activation of UPR^mt^. It is unclear why ATF5 did not translocate. In our recent study, application of a PERK inhibitor suppressed ATF4 nuclear localization, thereby preventing its suppression of *MMP2* gene expression (57). Due to the similarity between ATF4 and ATF5, changes in ATF5 phosphorylation status may be crucial for its nuclear localization. Indeed, ATF5 was recently demonstrated to be phosphorylated by Nemo-like kinase (58). Therefore, further investigation is required to explore the regulatory mechanisms involved in ATF5 nuclear localization.

To conclude, we provide evidence of UPR^mt^ activation in the placenta from cases of early-onset PE and have elucidated potential mechanisms for the UPR^mt^ pathway in the modulation of mitochondrial OXPHOS capacity. We also provide evidence of an additional regulatory mechanism of the UPR^mt^ pathway through PERK/eIF2α arm of UPR^ER^ pathway. A summary diagram is presented in *SI Appendix*, Fig. S8. Therefore, targeting cellular UPR pathways, both UPR^ER^ and UPR^mt^, could provide a new therapeutic intervention for early-onset preeclampsia. For example, the taurine conjugated bile acid, tauroursodeoxycholic (TUDCA), is being tested to alleviate the UPR^ER^ in diabetes.

## Materials and Methods

*SI Appendix*, *Materials and Methods* includes descriptions of the following items: chemicals and reagents; cell culture; rHR; RNAi knockdown of genes; mitoTracker Red staining and confocal microscopy; immunofluroscence; electron microscopy; quantitative real-time RT-PCR; and RNA sequencing, immunoblot analysis, and subcellular fractionation.

### Study Population and Placental Sample Collection.

The placental samples were obtained from the Research Centre for Women’s and Infants’ Health BioBank at the Lunenfeld-Tanenbaum Research Institute at Mount Sinai Hospital, University of Toronto, in conjunction with the hospital’s Placenta Clinic. Eligible subjects were invited to participate in the study and provided written informed consent. This study was reviewed and approved by the Human Subjects Review Committee of Mount Sinai Hospital (MSH REB no. 10-0128-E). PE was defined as new-onset hypertension (≥140/90 mmHg) observed on at least 2 separate occasions, 6 h or more apart, combined with proteinuria (a 24-h urine sample showing ≥ 300 mg/24 h). Only placentas from early-onset cases (<34 wk) were used in the study. One control group (NTC) was from healthy normotensive term patients that displayed no abnormalities on routine ultrasound examination. All preeclamptic and NTC placentas studied were delivered by nonlabored caesarean section. Another normotensive preterm control group (NPTC) was collected from pregnancies complicated by conditions including acute chorionic vasculitits, acute chorioamnionitis, and acute funisitis. These placentas were delivered vaginally. Women who smoked cigarettes or had chronic hypertension, diabetes mellitus, or preexisting renal disease were excluded.

For each placenta, 4 to 6 small pieces of tissue from separate lobules were rinsed 3 times in saline, blotted dry, and snap-frozen in liquid N_2_ within 10 min of delivery; the samples were stored at −80 °C.

The cryopreservation of placental tissues for mitochondrial respirometry were described previously (29). In brief, 3 pieces of villous samples (∼10 mg each) were biopsied from placentas, washed in PBS, and immersed in 200 μL of cryopreservation medium containing 0.21 M mannitol, 0.07 M sucrose, and 30% DMSO, pH 7.0) and allowed to permeate for 30 s before being snap-frozen in liquid N_2_ and transferred to −80 °C storage until later analysis.

### Mitochondrial Respirometry.

Respirometry was performed using Clark-type oxygen electrodes as described previously (29, 59).

#### Placental tissues.

Placental samples from 7 normotensive term control (NTC) and 12 early-onset PE (PE < 34 wk) were used for respirometry study and their clinical characteristics are presented in Table 1. As expected, there were significant differences between the 2 groups in gestational age at delivery, systolic and diastolic blood pressures, and placental and birth weights.

Before respirometry, the frozen placental tissue was thawed by mixing with prewarmed thawing medium (45 °C) containing 0.25 M sucrose and 0.01 M Tris⋅HCl, pH 7.5, at a ratio of 4:1 (medium:tissue) and incubated in a 45 °C water bath for ∼20 s. Immediately upon thawing, the tissues were transferred to tubes containing chilled BIOPS buffer containing 10 mM EGTA buffer, 0.1 mM free calcium, 20 mM imidazole, 20 mM taurine, 50 mM K-MES, 0.5 mM DTT, 6.56 mM MgCl_2_, 5.77 mM ATP, and 15 mM phosphocreatine, pH 7.1. The placental tissue was permeabilized in 1 mL of BIOPS containing 250 μg/mL saponin for 20 min at 4 °C with continual mixing. Tissues were washed twice for 5 min at 4 °C in respiration buffer (0.5 mM EGTA, 3 mM MgCl_2_, 60 mM C_12_H_21_KO_12_, 20 mM taurine, 10 mM KH_2_PO_4_, 20 mM Hepes, 110 mM sucrose, and 1 mg/mL BSA, pH 7.1) with continual mixing. The tissue was then ready for respirometry.

For respirometry, 30 mg of cryopreserved/thawed placenta were placed in a water-jacketed oxygen electrode chamber (MitoCell) at 37 °C (Strathkelvin Instruments Ltd) equilibrated to atmospheric O_2_ and the chamber was sealed. After permeabilization of the plasma membrane with saponin, LEAK state respiration rates were first acquired in the presence of 10 mM glutamate and 5 mM malate (GM_*L*_), before OXPHOS state respiration was stimulated by the addition of 2 mM ADP (GM_*P*_). At this point, cytochrome *c* was added to check for mitochondrial membrane integrity (10 mM). Next, complex I was inhibited by the addition of 0.5 µM rotenone and 10 mM succinate was added and OXPHOS respiration related to complex II (S_*P*_) was recorded. Electron transport was then inhibited at complex III by addition of 5 µM antimycin A. Complex IV-supported respiration (TmAs_*P*_) was stimulated by addition of 0.5 mM TMPD and 2 mM ascorbate, and oxygen consumption induced by auto-oxidation was assessed after inhibition of complex IV by sodium azide (100 mM). Subtraction from the rate of oxygen consumption prior to azide addition gave the complex IV supported respiration. Finally, placental fragments were removed from the electrode chambers, blotted, and dried for 48 h at 80 °C to obtain dry weights. RCR was calculated as the ratio of ADP-coupled respiration (GM_*P*_), which is the rate of oxygen consumption in the phosphorylated state after addition of ADP (GM_*P*_), divided by the rate of oxygen consumption in the presence of substrates without ADP (GM_*L*_), referred to as leak respiration. RCR reflects the coupling efficiency of ADP-stimulated respiration and can be used to assess mitochondrial membranes integrity.

#### BeWo-NG cells.

In brief, cells were trypsinized with 0.05% trypsin-EDTA (Thermo Fisher Scientific). The cell pellet was resuspended in BIOPS buffer containing 0.5 mM EGTA, 3 mM MgCl_2_0⋅6H_2_O, 20 mM taurine, 10 mM KH_2_PO_4_, 20 mM Hepes, 1 mg/mL BSA, 60 mM potassium-lactobionate, 110 mM mannitol, and 0.3 mM DTT, pH 7.1). Density of cell suspensions was determined using a hemocytometer and 10^6^ cells were added to a final volume of 500 µL respiratory medium containing 0.5 mM EGTA, 3 mM MgCl_2_, 60 mM C_12_H_21_KO_12_, 20 mM taurine, 10 mM KH_2_PO_4_, 20 mM Hepes, 110 mM sucrose, and 1 mg/mL BSA, pH 7.1, and transferred to the MitoCells at 37 °C. Cell membranes were selectively permeabilized with saponin (50 µg/mL) for 5 min, before mitochondrial respiration was measured. A substrate/inhibitor titration was used. Initially, 10 mM glutamate and 5 mM malate were added to the chambers, and LEAK state respiration was recorded (GM_*L*_). OXPHOS state respiration was stimulated by the addition of 2 mM ADP (GM_*P*_). Next, complex I was inhibited by the addition of 0.5 µM rotenone, before 10 mM succinate was added and OXPHOS respiration recorded (S_*P*_). Electron transport was then inhibited at complex III by addition of 5 µM antimycin A. Complex IV-supported respiration (TmAs_*P*_) was stimulated by addition of 0.5 mM TMPD and 2 mM ascorbate, and oxygen consumption induced by auto-oxidation was assessed after inhibition of complex IV by sodium azide (100 mM). Subtraction from the rate of oxygen consumption prior to azide addition gave the complex IV-supported respiration.

Between experiments using either placental tissue or BeWo-NG cells, oxygen electrode chambers were washed for at least 60 min with 100% ethanol and then several times with water to remove any trace of respiratory inhibitors.

### Statistical Analysis.

Differences were tested using a number of statistical analyses, including 2-tailed paired Student *t* test, 1-way ANOVA with Holm–Sidak’s multiple comparisons test or 2-way ANOVA with Tukey’s multiple comparison test according to the experimental design with *P* ≤ 0.05 considered significant. Correlations between proteins or genes were tested using the Pearson correlation, with *P* ≤ 0.05 considered significant. Power regression lines were fitted to display the relationship with *R*^2^ value. All statistical analyses were performed using GraphPad Prism v 6.0.

## Supplementary Material

Supplementary File
