# Supplementary material for: Noncanonical mitochondrial unfolded protein response impairs placental oxidative phosphorylation in early-onset preeclampsia
Source: Proc Natl Acad Sci U S A. 2019 Aug 22;116(36):18109–18. doi: 10.1073/pnas.1907548116 (PMC6731647; doi:10.1073/pnas.1907548116)
Supplement: Supplementary File [file pnas.1907548116.sapp.pdf]

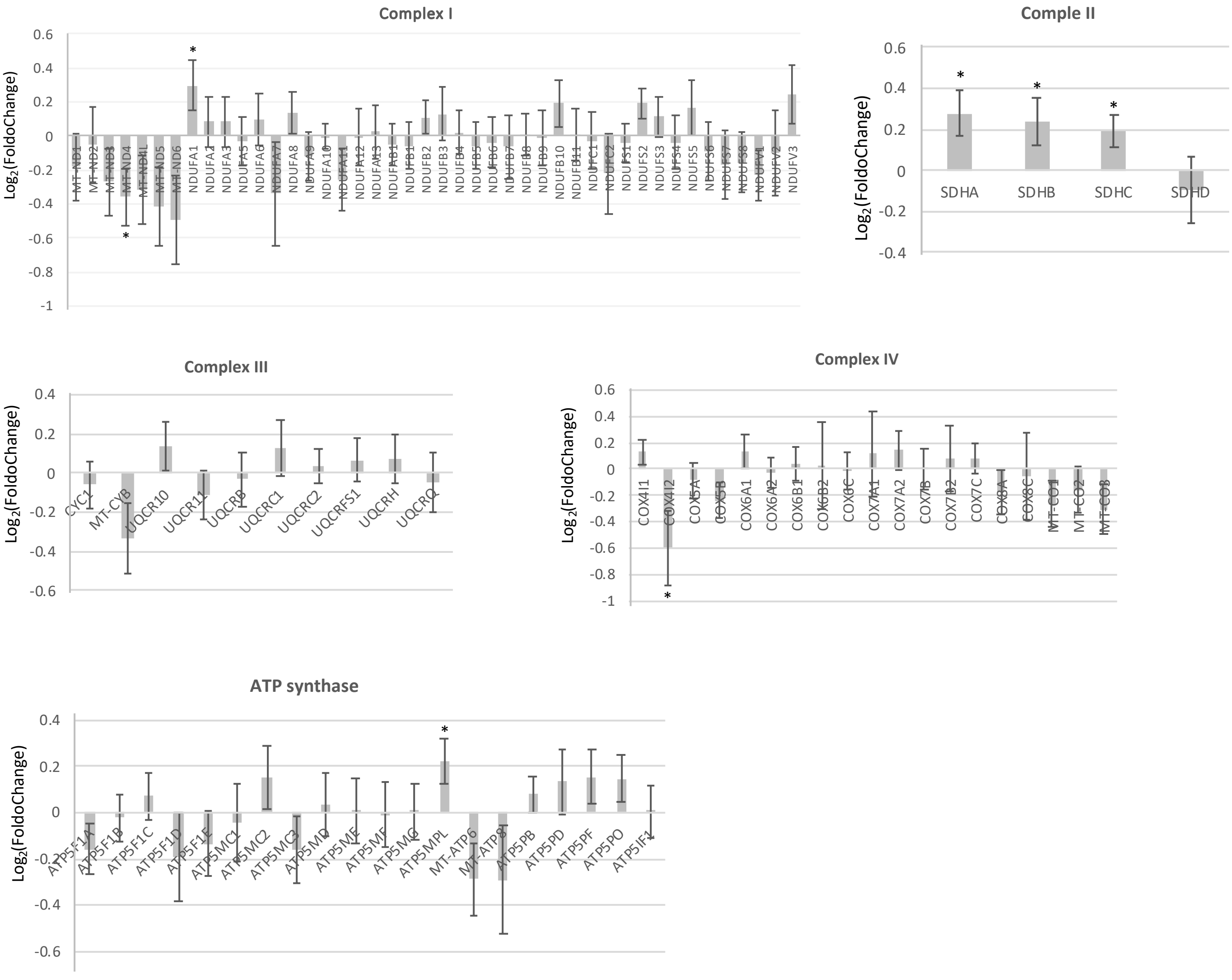


**Supporting figure 1 : Subtle changes in gene expression of all 97 mitochondrial ETC complexes subunits in the PE<34 wk placentas.** RNA was isolated for RNA-seq with NextSeq 75 high output run. Data are presented as mean±SEM, n=5-6. * P<0.05.

www.pnas.org/cgi/doi/10.1073/pnas.1907548116

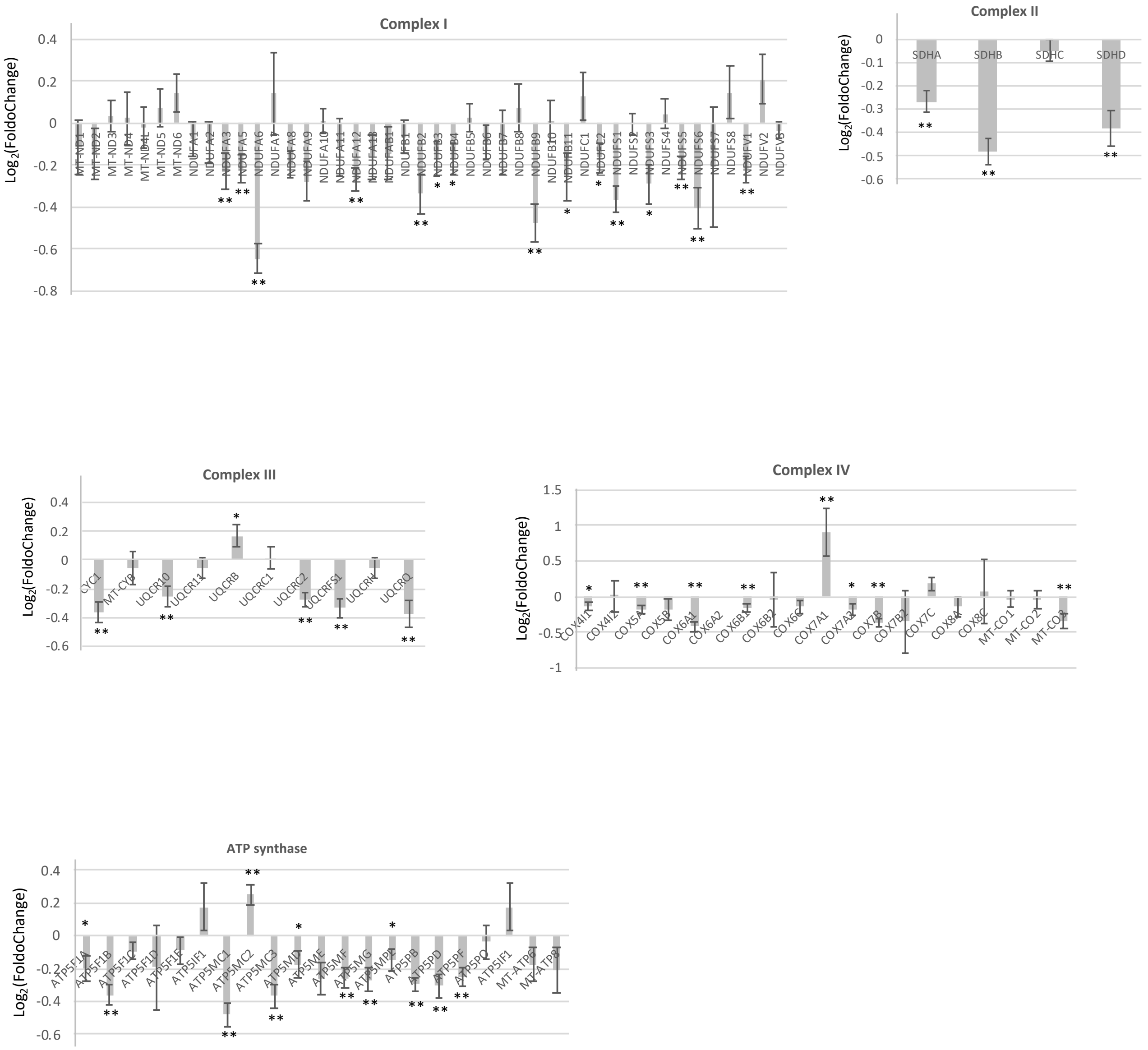


**Supporting figure 2 : Limited fluctuation of mitochondrial ETC complex subunits gene expression.** BeWo cells were subjected to rHR for 48 h. RNA was isolated and RNA-seq performed. All data are presented as $\log_2$(fold of change) in mean±SEM, n=5. * P<0.05; ** *P<0.01*. Half of the genes did not show significant change and only 4 genes showed over 30% change (*P<0.01*).

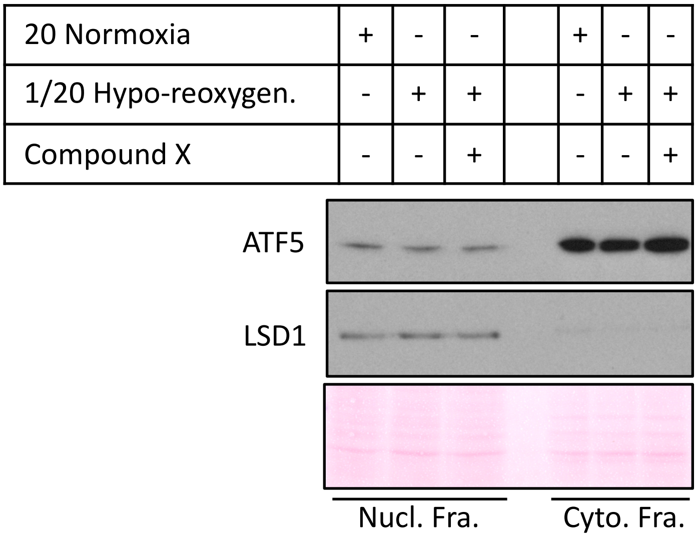


**Supporting figure 3 : No nuclear translocation of ATF5 in BeWo cells in response to rHR.** Cells were subjected to subcellular fractionation after 48 h rHR incubation. The enriched fractions of different cellular compartments were analysed by Western blotting. LSD1 was used as a nuclear marker. Ponceau S staining was used as a loading control.

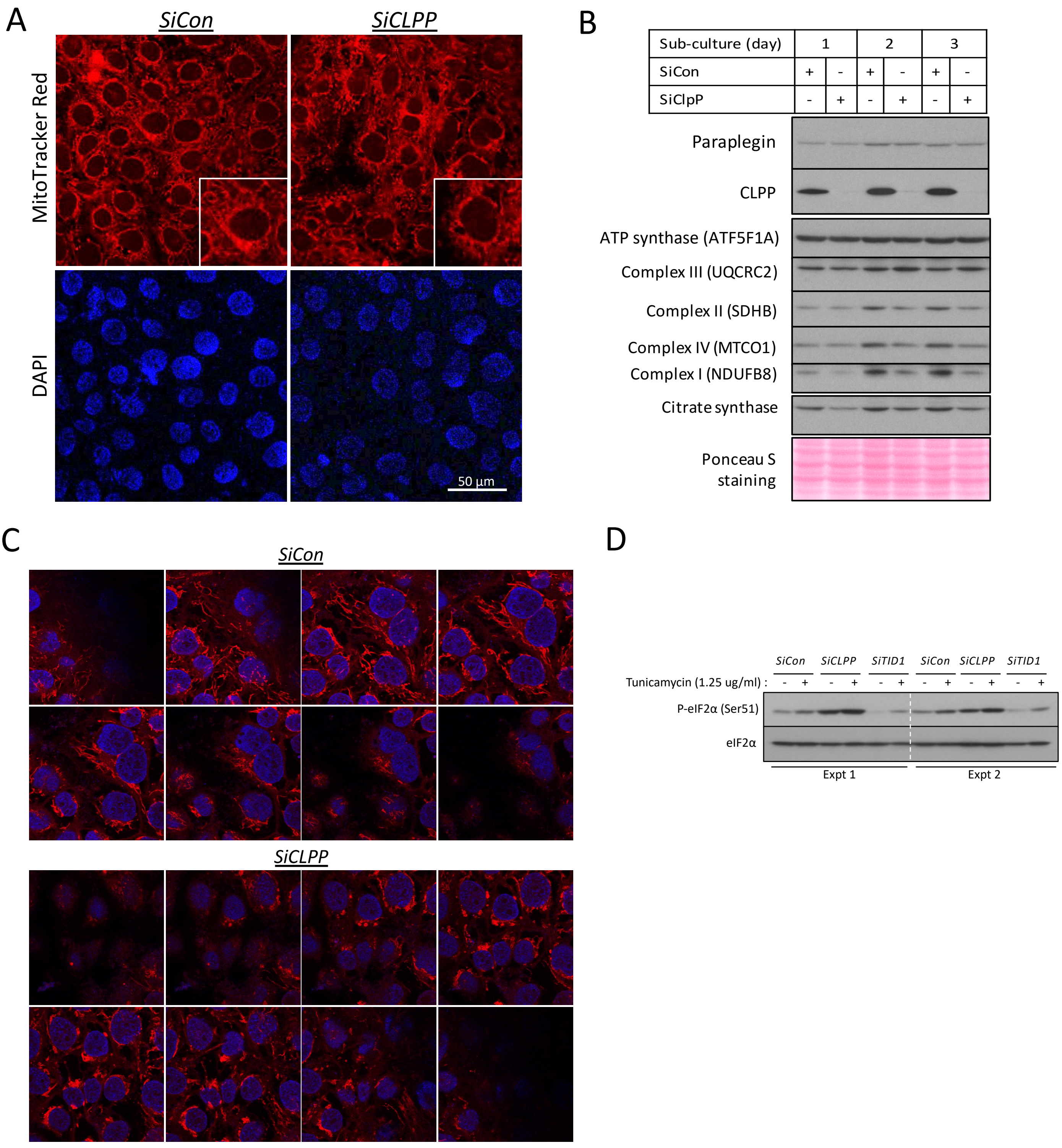


**Supporting figure 4 : Short-term knockdown down of CLPP does not affect mitochondrial membrane potential while prolonged suppression reduces mitochondrial ETC complexes subunit expression and facilitates mitochondrial fission. A)** No change in mitochondrial membrane potential after 48 h of transfection. **B)** After an additional 3 day subculture, *SiCLPP*-transfected cells showed persistent suppression of CLPP proteins. **C)** Change in mitochondrial morphology in *SiCLPP*-transfected cells is not an artefact from orientation of mitochondria. Images show serial Z-sections obtained by confocal microscopy. **D)** Knockdown of *CLPP* sensitizes the cells to ER stress, while down-regulation of *TID1* gene increases their resistance. RNA silencing was used to knockdown *CLPP* and *TID1* gene respectively in BeWo cells for 48 h prior to tunicamycin treatment. Cell lysates were harvested for western blotting to detect eIF2α activation.

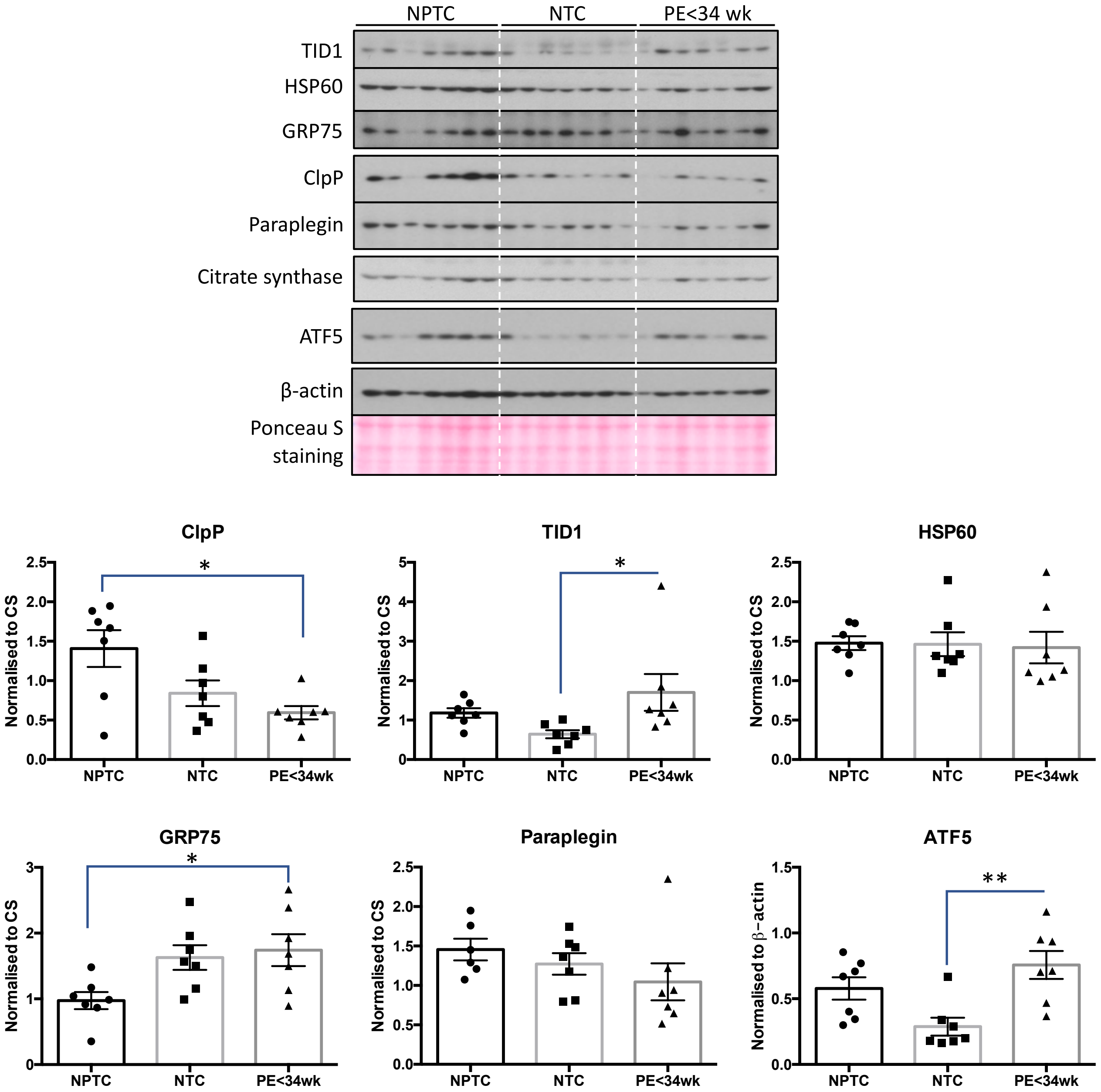


**Supporting figure 5: Level of UPR$^{mt}$ biomarkers in placentas of pre-term normotensive controls, term normotensive controls and early-onset preeclampsia.** Placental lysates were analyzed by western blotting with antibodies specific against TID1, HSP60, GRP75, CLPP, Paraplegin, ATF5 and β-actin. Both β-actin and ponceau S staining were used as loading controls. Band intensity was quantified and expressed as mean±SEM, n=7. Statistical analysis was carried out by one-way ANOVA with Tukey's multiple comparisons test. *P<0.05; **P<0.01.

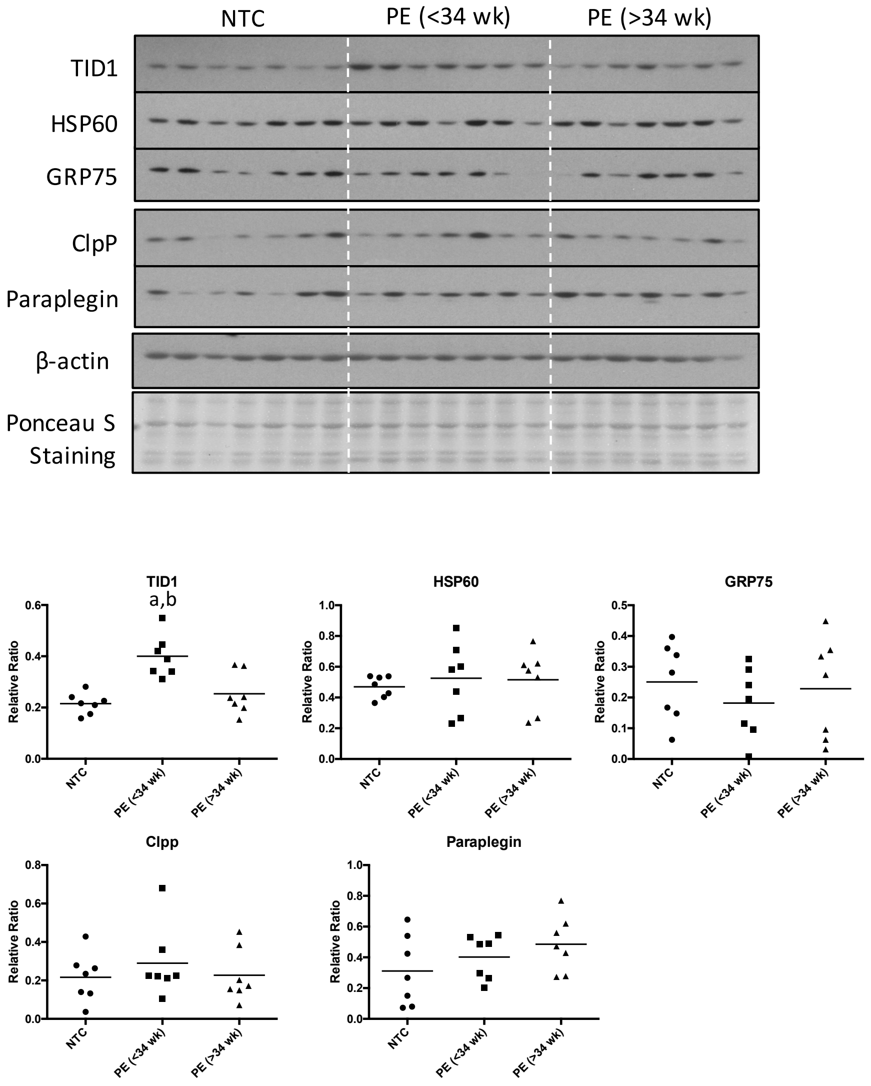


**Supporting figure 6 : No change of UPR$^{mt}$ biomarkers in placentas from late-onset pre-eclampsia.** Placental lysates were analyzed by western blotting with antibodies specific against TID1, HSP60, GRP75, CLPP, Paraplegin and ATF5. Band intensity was quantified and expressed as mean±SEM, n=7. Statistical analysis was carried out by one-way ANOVA with Tukey's multiple comparisons test. a & b indicate *P<0.05* compared to NTC and late-onset PE (PE >34 wk). (Clinical characteristics of this cohort are described in our previous publication, Yung et al.2014, J Path (23), 262-76).

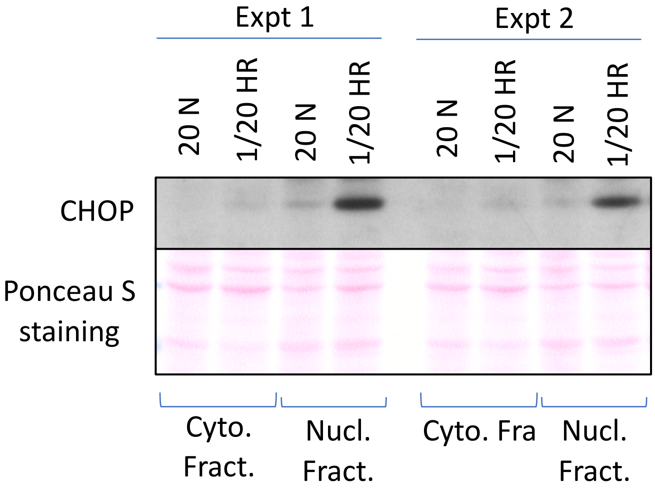


**Supporting figure 7: Increased nuclear translocation of CHOP after rHR challenge.** BeWo cells were challenged with rHR for 48 h before subjecting to nuclear fractionation. Western blotting was used to quantify the level of CHOP in nucleus and cytosol, and ponceau S staining was used to show protein loading of the samples.

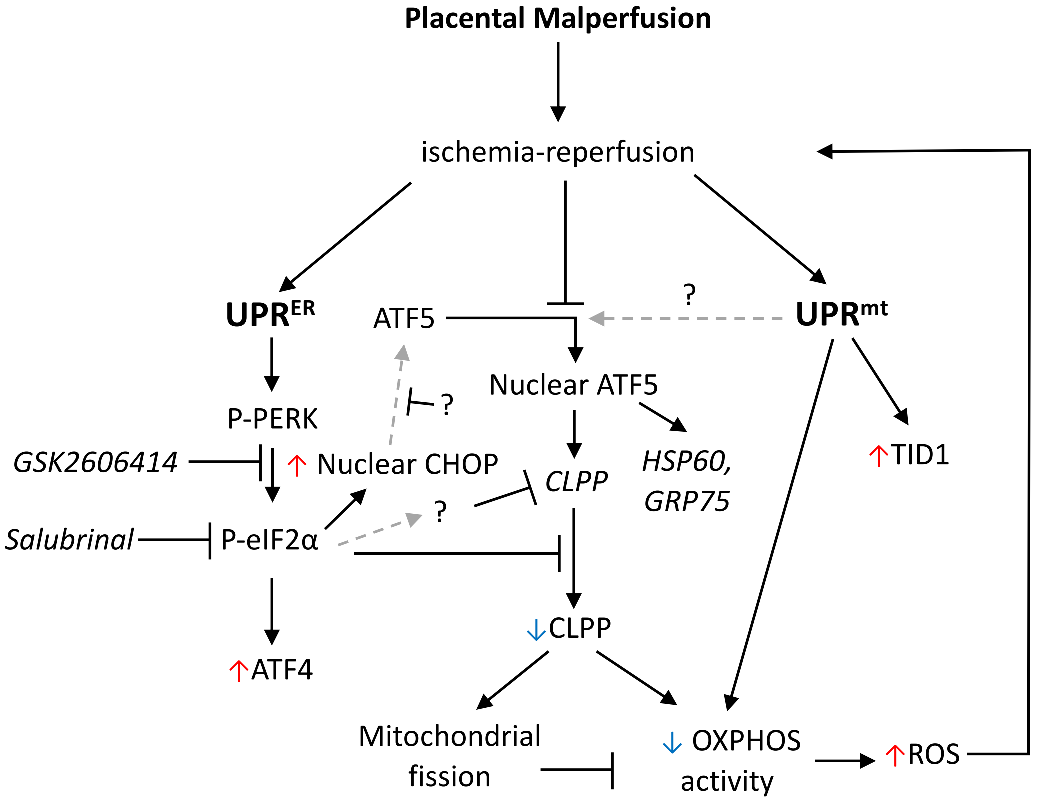


**Supporting figure 8: This diagram illustrates potential interactions between UPR$^{ER}$ and UPR$^{mt}$ signalling pathways in regulation of mitochondrial activity induced by placental malperfusion in early-onset preeclampsia.** In brief, placental malperfusion secondary to deficient remodelling of spiral arteries promotes ischemia-reperfusion injury to the placenta and activates both UPR$^{ER}$ and UPR$^{mt}$ signalling pathways which in turn reduces mitochondrial respiration by suppressing OXPHOS capacity. However, increased PERK-eIF2α activity of the UPR$^{ER}$ suppresses transcription and translation of the mitochondrial quality-control protease, CLPP, promotes mitochondrial fission and suppresses OXPHOS activity. The mechanism for transcriptional regulation of *CLPP* expression is unclear but is independent of ATF4 and CHOP. The nuclear localized CHOP fails to increase ATF5 protein indicating that an unknown mechanism(s) interact with CHOP in the regulation. It is also unclear why ATF5 does translocate into nucleus under hypoxia/ischemia-reperfusion, but no nuclear ATF5 may explain why there are no increases in the key mitochondrial chaperones, HSP60 and GRP75. Finally, loss of mitochondrial OXPHOS activity can facilitate ROS production, which can act as a positive feedback loop, thereby facilitating further oxidative damage. Arrow with dash grey line "  " and question mark " ? " indicate unknown pathway(s) & factor(s) involved in the regulation respectively.

*Supporting Information*

## Materials and Methods

All chemicals were from Sigma-Aldrich (Dorset, UK). Cell culture reagents were from Invitrogen (Paisley, UK).

## BeWo-NG cells Culture

BeWo-NG cells were generated by culturing BeWo cells at 5.5 mM physiological concentration of glucose as described previously (Yung et al., 2016). The cells were used at approximately >95 % confluence.

For treatment with tunicamycin, salubrinal and GSK2606414 (Generon, Slough, UK), cells were incubated with 1 mL (6 well plate) serum-free medium containing different concentrations of tunicamycin for 24 or 48 h.

For methacycline, cells were cultured in 2 mL (6 well plate) of medium containing 1 % FBS and different concentrations of methacycline for 24 or 72 h.

## Repetitive hypoxia-reoxygenation

BeWo-NG cells were subjected to cyclic fluctuating oxygen concentrations between 1 % and 20 % at 6 h intervals for 24 and 48 h in serum-free modified DMEM/F12 medium containing 5.5 mM glucose as described previously (5). Cells were cultured with 2 mL in humidified chambers with 5 % $CO_2$ at 37$^oC$ in an *Xvivo* System (BioSpherix Ltd, NY, USA).

## RNAi knockdown of *CLPP* and *ATF4* genes

RNA interference was performed using Lipofactamine® RNAiMax (Invitrogen) as described by the manufacturer. Cells were seeded at the density $10^5$ cells/mL in a 6-well plate a day before transfection to allow reaching ~30-40 % confluence. For transfection, 30 pmol of siRNA probe targeted against specific to either human *CLPP* (Sigma, cat no. SASI-Hs01_00025717 and SASI-Hs01_00025718) or *ATF4* (Sigma, cat no. SAS1_Hs02_00332313) was mixed with Lipofactamine before adding to the cells and left for 48 h before experiments. SiControl was performed using control siRNA sequences (Sigma, cat no. SIC001 and SIC002). The efficiency of transfection was checked by Western blot using antibodies against either CLPP or ATF4.

## MitoTracker Red staining and confocal microscopy

After experiments, mitochondrial membrane potential in BeWo-NG cells was measured by potential-sensitive MitoTracker Red CMXRos (ThermoFisher Scientific, Cheshire, UK) according to manufacturer instruction. In brief, the cells were incubated with 500 nM MitoTracker Red for 30 min before fixing with 4 % paraformaldehyde in PBS. After washing 3 times with PBS, cells were permeabilised with 0.1 % Saponin in PBS for 20 min before mounting with

Vectashield anti-fade medium with DAPI to the slide. Images were taken using a Leica TCS SP8 confocal microscope. Once optimized, the same PMT settings were used for both control and treated cells. The vertical z-axis sectioning was performed at 3 μm thickness.

**Immunofluorescence**

Cells were fixed with 4% paraformaldehyde in phosphate-buffered saline for 20 min prior to blocking with 1% bovine serum albumin and 0.1% saponin in phosphate-buffered saline. Primary antibody against ATF5 (Abcam, Cambridge, UK) was incubated overnight at room temperature following by incubation with secondary antibody conjugated with fluorescein Alexa 488 (Vector Laboratories, Peterborough, UK) for 1 h. The cells were stained in 5 μg/mL of Hoechst 33342 nuclear dye for 10 min. Images were taken by EVOS FL Color Imaging system (Life Technologies) at 200X magnification.

**Electron Microscopy**

Images were taken in the Cambridge Advanced Imaging Centre. In brief, small pieces of placental tissues were fixed by immersion in 2% glutaraldehyde containing 2 mM $CaCl_2$ in 0.1M PIPES buffer at pH 7.4; 100 μl 33% $H_2O_2$ was added to each 10 ml aliquot immediately before use. The tissues were fixed overnight at 4°C. They were rinsed twice in buffer (0.1 M PIPES) before post-fixation in 1% osmium ferricyanide for 1 h. After rinsing three times in distilled water they were stained in 2% uranyl acetate for 1 h and dehydrated in ascending concentrations to 100% ethanol. They were rinsed twice in acetonitrile, and embedded in Quetol epoxy resin. Fifty-nanometer sections were cut on a Leica Ultracut UCT, stained with saturated uranyl acetate in 50% ethanol and lead citrate, and viewed in a FEI Philips CM100 operated at 80 kv.

**Reverse Transcription and Quantitative real-time PCR**

Total RNA was isolated using an RNeasy mini kit (Qiagen, UK) according to the manufacturer's instructions. The concentration of RNA was determined using a NanoDrop™ spectrophotometer. The ratio of absorbance between 260 nm/280 nm for all samples was over 2.

For first-strand cDNA synthesis, 1 μg of total RNA was mixed with random hexamers and RNAase-free water and heated at 70°C for 10 min. Afterwards, reaction mixture containing first strand buffer, DTT, dNTPs (ThermoFisher, UK) and RNaseOut (ThermoFisher, UK) was added and incubated at room temperature for 10 min followed by incubation at 42°C for 2 min before adding SuperScript® III Reverse Transcriptase (ThermoFisher, UK). Afterwards, the reaction mixture was incubated at 42°C for an additional 50 min, followed by 65°C for 15 min.

qPCR was performed using SYBR Green JumpStart kits (Sigma, Dorset, UK) according to the manufacturer's instruction in thermocycler DNA Engine OPTICON2 (MJ Research, UK). The primer sequences were: *CLPP* forward 5'-

AGCTCTATAACATCTACGCC-3'; *CLPP* reverse 5'-CAGAACCTTGTCTAAGATGC-3', and two endogenous internal controls, *TBP* forward 5'-GTGGGGAGCTGTGATGTGA-3'; *TBP* reverse 5'AATAAGGAGAACAATTCTGGTTTG-3' and *18S* forward 5'-GTAACCCGTTGAACCCCATT-3'; *18S* reverse 5'-CCATCCAATCGGTAGTAGCG-3'. The PCR profile was at 95 $^{o}$C for 5 min following by 40 cycle of 95 $^{o}$C for 15 sec, 30 sec at 53 $^{o}$C (*CLPP*) and 61 $^{o}$C for both *18S* and *TBP*. The gene expression levels were calculated using the threshold cycle method ($2^{-\Delta\Delta CT}$ method) with reference to the average value of *18S* and *TBP*. The results were presented as relative expression levels.

**RNA Extraction and RNA Seq**

RNA Seq was carried out by Cambridge Genomic Services, University of Cambridge (UK). RNA was extracted from BeWo-NG cells using the RNeasy Plus Universal Mini Kit (Qiagen, UK) and RNA quality was checked by Agilent 2100 RNA analyser. Libraries were made using the Illumina TruSeq Stranded mRNA Library Kit according to the manufacturer's instructions. Libraries were quantified (kappa qPCR), and equimolar pools were sequenced (single end 50 base reads, SE50) in several lanes of the Illumina HiSeq2500.

**Tissue/cell lysate preparation and Western blot**

Seven placental samples per group from preterm normotensive control (PNTC), normotensive term control (NTC), and early-onset pre-eclampsia (PE<34 wk) were used for western blotting analysis and clinical characteristic of the 3 groups were presented in Table 2.

Both cells and placental tissues were prepared in lysis buffer containing 20 mM Tris (pH 7.5), 150 mM NaCl, 1 mM EDTA, 1 mM EGTA, 1% Triton X-100, 2.5 mM sodium pyrophosphate, 1 mM β-glycerolphosphate, 1 mM $Na_3VO_4$, and complete mini EDTA-free proteases inhibitor cocktail (Roche Diagnostics, East Sussex, UK). For tissues, they were homogenized with Lysing Matrix D (MP Biomedicals, UK) in MagNA Lyser Instrument (Roche Diagnotics Ltd, UK). Bicinchoninic acid kit (Sigma-Aldrich, UK) was used to determine protein concentration in the cell or tissue lysate.

Both cell and tissue lysates were prepared in gel loading buffer containing 50 mM Tris-HCl (pH 6.8), 100 mM DTT, 2% SDS, 10% glycerol following by heating at 70$^{o}$C for 10 min before loading and resolving in SDS-PAGE gels following by blotting onto nitrocellulose. Ponceau S solution (Sigma-Aldrich, UK) was used to stain the membrane as a loading control. The membrane was blocked with 5% skimmed milk in TBS-T following by primary antibody incubation overnight at 4$^{o}$C and a few hours at room temperature. The membrane was then incubated with HRP-conjugated secondary antibody (GE Healthcare, UK) for 1 h. The signal of resolved protein was analysed by enhanced chemiluminescence (ECL) (Amersham Biosciences, UK) using Kodak X-OMAT (AR) film (Sigma-Aldrich, UK). Films were scanned using a flat-bed scanner (HP Scanject G4050) and band intensities were determined from two or three different exposures (within the

linear detection range) with background subtraction using Image J software (Freeware from https://imagej.nih.gov/ij/download.html).

**List of primary antibodies and conditions for WB**

| Primary Antibody | Catalogue Number | Dilution | Incubating buffer | Company |
|---|---|---|---|---|
| CLPP | ab124822 | 1:1000 | TBS-T | Abcam |
| HSP60 | ab46798 | 1:4000 | TBS-T | Abcam |
| GRP75 | ab2799 | 1:4000 | TBS-T | Abcam |
| Citrate synthase | ab96600 | 1:2000 | TBS-T | Abcam |
| ATF5 | ab184923 | 1:1000 | TBS-T | Abcam |
| Phospho-eFI2α (Ser51)(D9G8) | 3398 | 1:1000 | TBS-T | Cell Signalling Technology |
| eIF2α | 2103 | 1:1000 | TBS-T | Cell Signalling Technology |
| ATF4 (D4B8) | 11815 | 1:1000 | TBS-T | Cell Signalling Technology |
| DRP1 | 8570 | 1:1000 | TBS-T | Cell Signalling Technology |
| OPA1 | 80471 | 1:1000 | TBS-T | Cell Signalling Technology |
| TID1/DnaJ/HSP40 | GTX111077 | 1:1000 | TBS-T | GeneTex |
| Paraglegin(C-5) | Sc-514393 | 1:500 | 5% skimmed milk | Santa Cruz Biotehnologies |
| Anti-Hu OXPHOS complex kit | 458199 | 1:1000 | TBS-T | Novex, Life Technologies |
| GRP78 | 610978 | 1:5000 | TBS-T | Transduction Lab |
| B-actin | A2228 | 1:10000 | TBS-T | Sigma-Aldrich |

## Subcellular Fractionation

After treatment, cells were scraped off and collected in PBS before centrifugation at 200 g for 5 min at 4$^o$C. The pellet was resuspended gently in hypotonic buffer containing 20 mM HEPES, pH7.9, 10 mM KCl, 0.1 mM EDTA, 0.1 mM EGTA, 1 mM DTT and protease inhibitors (Complete mini protease inhibitors cocktail, Roche), by pipetting up and down and incubated on ice for 15 min with occasional inversion. The cells were broken down using a Dounce homogenizer with 15 strokes on ice, and the homogenate centrifuged at 3,000 g for 5 min at 4$^o$C. The supernatant was transferred to a new tube and centrifuged at 12,000 g for 15 min at 4$^o$C to isolate mitochondrial and cytosolic fractions, while the crude nuclear fraction was resuspended in resuspension buffer containing 20 mM HEPES, pH 7.9, 400 mM NaCl, 1 mM EDTA, 1.5 mM $MgCl_2$, 1 mM DTT and protease inhibitors and incubate on ice for 30 min before centrifugation at 12,000 g for 10 min at 4$^o$C. The supernatant contained an enriched nuclear fraction.
